# Stable porous graphene oxide membranes enabled by confined growth of 2D MOF nanosheets for high-performance desalination

**DOI:** 10.1126/sciadv.aee2550

**Published:** 2026-05-15

**Authors:** Mengjiao Zhai, Farhad Moghadam, Guangrui Wang, Mohd Hafiz Dzarfan Othman, Kang Li

**Affiliations:** ^1^ Barrer Centre, Department of Chemical Engineering, Imperial College London, Exhibition Road, London SW7 2AZ, UK.; ^2^ Advanced Membrane Technology Research Centre, School of Chemical and Energy Engineering, Universiti Teknologi Malaysia, Skudai, Johor 81310, Malaysia.

## Abstract

Graphene oxide (GO) membranes hold substantial promise for this application but are limited by structural instability in aqueous environments. This study introduces a composite membrane based on porous graphene oxide (PGO) with two-dimensional copper 1,4-benzenedicarboxylate (CuBDC) nanosheets grown in situ. The confined growth of CuBDC within the PGO laminar structure, via strong coordination between Cu^2+^ ions and oxygen-containing groups on PGO, not only stabilizes the PGO laminar structure but also induces NaCl rejection due to the appropriate pore size of the CuBDC. The resulting composite membrane demonstrated a high-water flux of 124 kg m^−2^ hour^−1^ in conventional pervaporation and 89 kg m^−2^ hour^−1^ in low-energy water carrier pervaporation, with NaCl rejection consistently above 99.9%. Technoeconomic analysis reveals that desalination using the fabricated membrane in a water-carrier pervaporation process results in a low annual expenditure. Overall, this study offers a promising strategy for stabilizing PGO membranes with excellent selectivity, paving the way for more energy-efficient desalination technologies.

## Teaser

Confined 2D MOF growth in PGO nanochannels rich in reactive sites enhances membrane stability and desalination performance.

## INTRODUCTION

Two-dimensional (2D) graphene oxide (GO) and its derivatives, such as porous GO (PGO), are promising membrane materials because of their easy accessibility, chemical stability, and strong antifouling properties (*1*). A single GO nanosheet features the coexistence of sp^3^ domains hybridized with oxygen-containing groups and sp^2^ regions inherited from graphene (*2*). Self-assembled GO nanosheets can be obtained by stacking, leading to a laminar membrane structure. Theoretically, the abundant functional groups on the lamellar planes and edges of the nanolayers impart hydrophilicity, promoting the preferential adsorption of water molecules. Moreover, the nonoxide zones between adjacent GO nanosheets act as 2D capillaries that enable a near-frictionless flow of water while impeding the transport of larger hydrated species (*3*, *4*). Despite these advantages, the widespread deployment of GO membranes in aqueous environments remains significantly constrained by their inherent stability issues in aqueous solutions.

The oxygen-containing functional groups in GO membranes pose significant challenges to their structural stability in aqueous environments. These groups readily undergo hydration, generating negative charges that lead to strong electrostatic repulsion between adjacent layers. This repulsion can increase the interlayer spacing, diminish selectivity, and potentially cause delamination of the GO membrane within several hours (*5*). Extensive research has sought to mitigate the swelling effect through methods such as partial reduction (*6*), covalent crosslinking (*7*), and cross-linking with multivalent cations (*8*). GO reduction is typically achieved via chemical/photo treatments to remove oxygen-containing groups. However, it markedly decreases membrane permeability because of the smaller interlayer spacing in reduced GO (*9*). Covalent cross-linking with organic compounds is another method to improve GO membrane stability. This increases the interlayer d-spacing, enhancing the permeance but at the expense of reduced rejection (*5*). Cation-GO cross-linking, which is based on cation-π interactions and cation-oxidizing group interactions, can stabilize the GO structure with controlled interlayer spacing. However, the stability of this interaction is compromised by the leakage of cations when ions with stronger interactions are present (*8*). Therefore, innovative strategies to stabilize GO membranes without compromising the water permeation rate or size-sieving ability are urgently needed.

Stabilizing GO membranes through interactions with metal-organic frameworks (MOFs) has emerged as a promising strategy to increase structural integrity while selectively preserving separation performance. MOFs, synthesized via reticular chemistry, offer highly tuneable functionalities, enabling strong coordination with functional groups on GO to mitigate delamination and improve mechanical stability (*10*). Furthermore, the intrinsically porous architecture with a tailorable pore size is expected to increase mass transport pathways and enhance the molecular sieving capability of the resulting membranes (*11*). Alemayehu *et al.* (*12*) demonstrated the fabrication of an Al-MOF/GO composite membrane using a costacking strategy, achieving stable nanofiltration performance over an 80-hour continuous dead-end test. Owing to its simplicity, this approach has been widely used in the construction of GO/MOF composite membranes (*13*). Nevertheless, the costacking strategy relies on the independent synthesis of GO and MOFs, followed by their vacuum-assisted assembly. When coordination interactions between metal ions and functional groups on GO are realized after assembly, the dominant interfacial forces are considered as the electrostatic interactions and π-π stacking due to the intrinsic stability of MOFs after synthesis (*12*, *13*). This inherently weak interlayer adhesion significantly undermines the stabilization efficacy of MOFs within GO membranes, thereby limiting their long-term durability. Moreover, the vacuum-assisted stacking process leads to a random distribution of MOFs within the GO membrane, resulting in a failure to selectively patch lateral defects with the intrinsically porous architecture of the MOFs (*14*).

The in situ growth of MOFs within GO membranes represents a highly promising strategy for establishing robust coordination interactions. Recently, Moghadam *et al.* (*15*) reported the fabrication of a hybrid PGO/zeolite imidazolate framework-8 (ZIF-8) membrane through a two-step in situ growth approach, achieving targeted coordination between Zn^2+^ from ZIF-8 and hydroxyl functional groups on GO. This precise coordination not only imparted good structural robustness, enabling the membrane to withstand a 50-hour continuous crossflow test, but also enhanced its molecular selectivity. Despite this advancement, the incorporation of 3D MOFs within the laminar framework of GO has raised fundamental concerns (*16*). The laminar structure of the GO membrane is particularly advantageous for mass transport in nonpressure-driven membrane separation processes, such as pervaporation (PV) and membrane distillation. In these systems, the narrow nanochannels between 2D laminae generate substantial capillary pressure once the liquid/vapor interface is established. This provides a key driving force for the efficient transport of water across a large portion of the membrane’s nanochannels (*17*). However, the integration of MOFs with a 3D configuration inevitably disrupts the ordered laminar framework of GO, significantly compromising structural uniformity and hence affecting capillary pressure. To address this limitation, tailoring the in situ growth of 2D MOF nanosheets represents a compelling strategy for enhancing GO membrane stability while preserving its advantageous laminar structure. However, achieving this goal remains challenging because of the lack of precise control over targeted nucleation and oriented growth kinetics of 2D MOFs within the interlayer space of GO membranes.

**Fig. 1.**
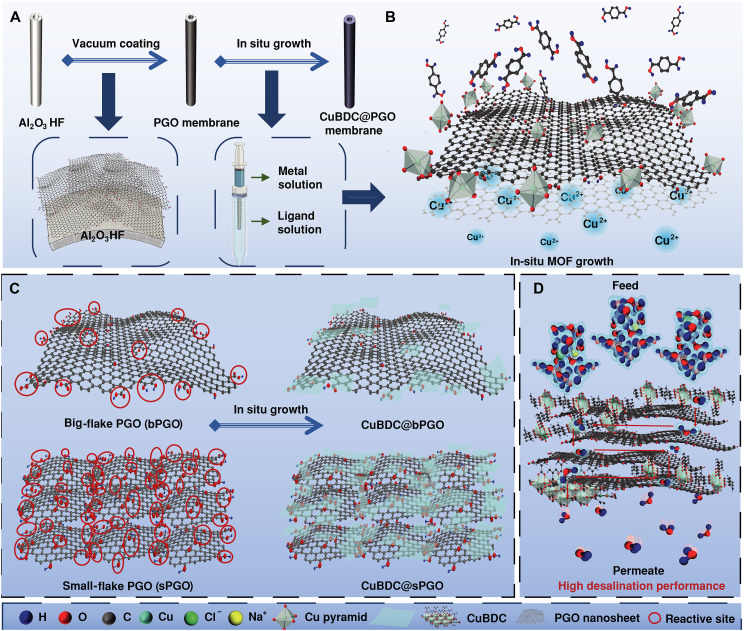
Schematic illustration of the membrane fabrication and desalination process. (**A**) Fabrication process of the CuBDC@PGO composite membrane. (**B**) CuBDC–PGO interaction via coordination between oxygen-containing groups and the Cu pyramid during the contra diffusion step. (**C**) Schematic illustration of the increase in reactive sites resulting from the fragmentation of bPGO into sPGO and the corresponding increase in CuBDC loading. (**D**) Desalination using the CuBDC@PGO composite membrane. The red arrows indicate the mass transport pathways through the composite membrane.

In this work, we fabricate a MOF@GO composite membrane by confined growth of a water-stable 2D MOF, copper 1,4-benzene dicarboxylate (CuBDC) (*18*), in the pore edges and within the laminar structure of a PGO membrane (Fig. 1). CuBDC nanosheets are synthesized at the pore edges and within the laminar structure of a vacuum-coated PGO hollow fiber (HF) membrane via a contradiffusion method (Fig. 1, A and B). The oxygen-containing groups of the PGO nanosheets highlighted with red circles in Fig. 1C offer reactive sites to coordinate with metal ions, promoting the in situ formation of metal clusters and facilitating robust interactions between the MOF and PGO (Fig. 1B). The CuBDC loading significantly increased when large-flake PGO was fragmented into small-flake PGO for membrane fabrication owing to the increased density of reactive sites (Fig. 1C). The targeted coordination of reactive sites with CuBDC significantly enhances the structural robustness of the composite membrane, thereby stabilizing the PGO membrane during desalination tests conducted via both reverse osmosis (RO) and PV (Fig. 1D). The preservation of well-defined 2D capillaries enables an excellent flux of ~124 kg m^−2^ hour^−1^ under conventional PV and a high flux of 89 kg m^−2^ hour^−1^ in a low-energy water-carrier PV (WCPV) system (fig. S1). A comprehensive technoeconomic analysis (TEA) further highlights the strong potential of the fabricated membrane for large-scale hypersaline water treatment with reduced energy consumption. Overall, this study establishes a favorable interfacial platform that regulates nucleation and crystal growth, thereby offering a promising strategy to enhance both the stability and performance of 2D frameworks in molecular separation technologies.

## RESULTS

### Membrane fabrication and characterization

**Fig. 2.**
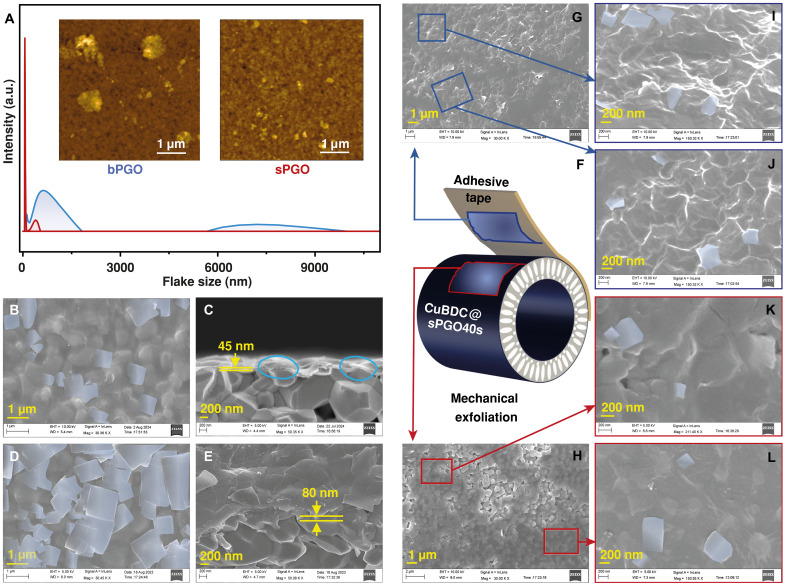
PGO flake sizes and morphologies of the pristine and exfoliated CuBDC@PGO membranes. (**A**) AFM image of bPGO and sPGO flakes and the flake size distributions of bPGO and sPGO nanosheets measured via DLS. Surface and cross-sectional morphologies of (**B** and **C**) CuBDC@bPGO40s and (**D** and **E**) CuBDC@sPGO40s. (**F**) Mechanical exfoliation with the assistance of an adhesive tape and the inner microstructure of (**G**) the membrane adhered to the tape and (**H**) the membrane remained on the HF surface. Regions outlined in blue or red frames are shown at higher magnification (**I**, **J**, **K**, and **L**), as indicated by the corresponding arrows. a.u., arbitrary units.

PGO nanosheets with atomically thin 2D structures readily self-assemble into layered structural films under vacuum filtration (Fig. 1A). Two types of PGO flakes were used for vacuum coating: big-flake PGO (bPGO) (*19*), with a size range of 0.3 to 1.6 μm, and small-flake PGO (sPGO), obtained by fragmenting bPGO through a facile tip sonication process, with a size range of 0.2 to 0.4 μm (Fig. 2A). The membranes were prepared with coating times of 40, 80, or 120 s, denoted bPGO40s/80s/120s and sPGO40s/80s/120s, respectively. The subsequent CuBDC loading was achieved via in situ growth using a contradiffusion technique, in which a copper (II) nitrate trihydrate solution (metal precursor) and a terephthalic acid solution (organic ligand) were introduced to the lumen and shell sides of the PGO-HF membrane, respectively. The Cu^2+^ ions initially formed pyramidal clusters coordinated by five oxygen atoms. These clusters subsequently coordinated with terephthalate carboxyl groups (Fig. 1B), leading to the in situ formation of CuBDC within the PGO membrane. The oxygen-containing groups on the PGO basal planes play a dual role in this process: either directly participating in metal cluster formation or coordinating with preformed clusters, both of which facilitate subsequent MOF growth (*14*, *20*).

Scanning electron microscopy (SEM) revealed the surface and cross-sectional structures of the CuBDC@bPGO membranes (Fig. 2B and fig. S2). The CuBDC nanosheets on bPGO (highlighted in light blue) displayed tetragonal or near-tetragonal shapes with lateral dimensions of 0.4 to 2.0 μm. Square-edged nanosheets were also evident in cross sections (blue circles, Fig. 2C and fig. S2), confirming successful CuBDC growth in the bPGO membrane.

The CuBDC loading in the composite membrane was greater in the sPGO-based membranes because of the increased number of reactive sites. In the sPGO membranes, the CuBDC nanosheets with a uniform tetragonal morphology covered nearly the entire surface (Fig. 2D and fig. S3), far more extensively than those in the bPGO membranes did despite the pristine bPGO and sPGO showing nearly identical morphologies (figs. S4 and S5). The increased CuBDC loading was also evident from the increased membrane thickness (Fig. 2E and fig. S3). The CuBDC@bPGO composite membrane exhibited a 12.5 to 15.4% increase in thickness relative to that of the pristine bPGO membrane, whereas the CuBDC@sPGO composite membrane displayed a substantially larger increase of 50 to 60%, indicating higher MOF loading in the sPGO-based membranes (table S1). The increased MOF loading is attributed to the increased number of reactive sites. As illustrated in Fig. 1 (B and C), the oxygen-containing functional groups that coordinate with the MOF are located primarily at the edge and defect sites of the PGO layer (*21*). Reducing the flake size, as confirmed by atomic force microscopy (AFM) and dynamic light scattering (DLS) (Fig. 2A), increased the number of exposed edges per unit area, supplying more coordination sites and thus promoting higher loading. This controllable strategy for tuning MOF loading provides a promising route for tailoring membrane structure and regulating separation performance.

The formation of the CuBDC@PGO composite membrane was further verified by elemental mapping. As shown in fig. S6, carbon was uniformly distributed in all the samples, whereas copper appeared only in the CuBDC@PGO membranes, with a notably greater density in the CuBDC@sPGO membranes.

SEM also confirmed the confined growth of 2D CuBDC nanosheets within the PGO laminar framework. As shown in Fig. 2F, a mechanical exfoliation method with the assistance of adhesive tape was used to reveal the inner microstructure of the CuBDC@sPGO40s membrane. After exfoliation, the membrane was partially stuck to the tape (Fig. 2G), while the remainder remained on the HF surface (Fig. 2H), exposing the inner composite structure. The CuBDC nanosheets were found to be well aligned along the inner sPGO basal planes (highlighted in light blue) from both the tape-adhered layers (Fig. 2, I and J) and the HF-retained layers (Fig. 2, K and L). Notably, these internally confined nanosheets exhibit a lower density and smaller lateral dimensions (~0.2 to 0.6 μm) compared with the nanosheets formed on the membrane surface. In contrast, CuBDC crystals synthesized directly from the corresponding metal and ligand solutions display a broad lateral size distribution and a densely stacked lamellar morphology (fig. S7, A and B) (*18*). This observation provides strong evidence of the confined growth of the CuBDC nanosheets within the PGO laminae.

The confined growth of 2D CuBDC effectively preserved the laminar architecture in both the high-MOF–loaded CuBDC@sPGO and low-MOF–loaded CuBDC@bPGO membranes, as evidenced by the cross-sectional images shown in Fig. 2 (C and E) and figs. S2 and S3. This structural preservation ensures membrane integrity and robustness. This outcome underscores the effectiveness of the growth method used in this study. Here, the PGO layer served both as a loading platform, providing the reactive site for MOF loading, and as a barrier that prevented bulk CuBDC formation. In the absence of the PGO confinement layer, randomly oriented CuBDC lamellae with lateral dimensions more than an order of magnitude larger were observed on the substrate (fig. S8). The proposed strategy effectively confined CuBDC intergrowth by combining the limited nutrient supply characteristic of the contradiffusion technique with the controlled adjustment of reactive sites in the sp^2^ region (*22*). Furthermore, the interlayer spacing of PGO in the CuBDC synthesis precursor was estimated to be ~1.25 to 1.6 nm (*23*), providing a spatial constraint that further confines the CuBDC growth along the 2D channels. These factors collectively ensure the preservation of the laminar architecture, which is critical for maintaining the functionality and stability of the membrane.

Composite membranes were also fabricated using vacuum-assisted coating and conventional in situ growth. The vacuum-coated membrane (fig. S9A) exhibits loosely stacked, randomly aligned layers with visible interflake gaps, reflecting imperfect self-assembly. In contrast, the membrane (fig. S9B) prepared by direct in situ growth shows sparsely distributed, bulk-like aggregates primarily on the membrane surface, resulting from rapid homogeneous nucleation upon direct mixing of metal and ligand solutions. These observations further demonstrate the advantage of the contradiffusion strategy in enabling confined and regulated growth of 2D CuBDC nanosheets within the PGO laminar framework.

**Fig. 3.**
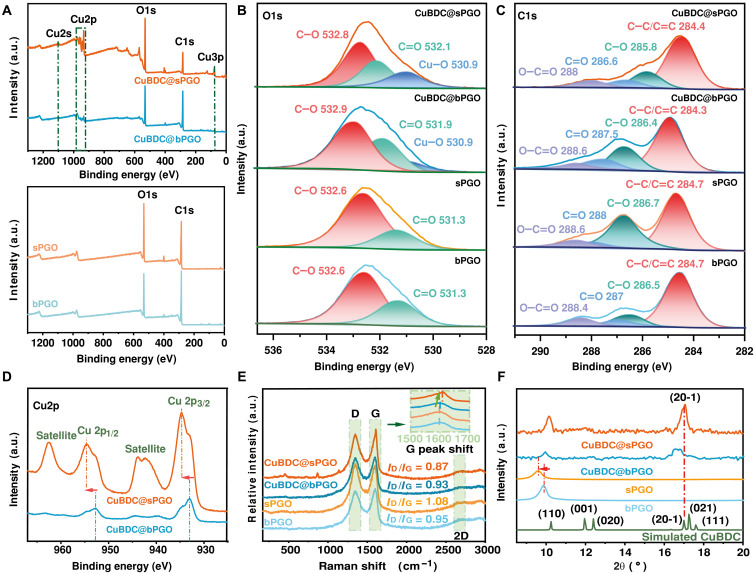
Structural characterization of the membrane surfaces. (**A**) Full-range XPS spectra. (**B** to **D**) High-resolution XPS spectra of (B) O1s, (C) C1s, and (D) Cu2p of the bPGO40s, sPGO40s, CuBDC@bPGO40s, and CuBDC@sPGO40s membranes. (**E**) Three characteristic peaks are observed: the D band, indicating the presence of structural defects such as disorder, vacancies, and edges; the G band, corresponding to sp^2^-hybridized carbon domains; and the 2D band, arising from the in-plane breathing modes of the carbon rings. The inset in (E) provides an enlarged view of the spectral region between 1500 and 1700 cm^−1^ for all the samples. (**F**) XRD patterns of the simulated CuBDC and bPGO40s, sPGO40s, CuBDC@bPGO40s, and CuBDC@sPGO40s membranes.

The x-ray photoelectron spectroscopy (XPS) spectra shown in Fig. 3, A to D reveal the elemental composition and bonding interactions within the membranes. The full-range spectrum (Fig. 3A) showed a stronger O1s peak in sPGO, with the oxygen content increasing from 25.44 to 29.38 atomic % (at %) (table S2). The Raman spectra (Fig. 3E) corroborated this finding, showing an elevated intensity ratio of the D band to the G band (*I*_D_/*I*_G_) from 0.95 to 1.08, indicating that more sp^3^-hybridized domains that act as reactive sites for CuBDC coordination. Consequently, the CuBDC@sPGO membrane has a significantly greater copper content (21.5 at %), nearly four times greater than that of CuBDC@bPGO (5.03 at %), as shown in table S2. A weaker Cu signal on random sites of exfoliated CuBDC@sPGO40s (fig. S10) further indicated confined growth within the PGO layers.

High-resolution XPS spectra [Fig. 3 (B to D)] confirmed the interaction between the CuBDC and PGO membranes. As shown in Fig. 3B, the O1s spectra of the composite membranes present three characteristic peaks, with a new peak at 530.9 eV corresponding to O anions in the crystalline CuBDC network (*24*). This peak was stronger for CuBDC@sPGO than for the other samples, suggesting enhanced coordination between the Cu and oxygen functional groups. In addition, shifts in C═O (from 531.3 to 532.1 eV) and C─O (from 532.6 to 532.8 eV) for PGO imply electron transfer from oxygen-containing groups to Cu(II), confirming coordination (*21*). These findings support our hypothesis that the metal clusters of CuBDC interact with the PGO membrane through coordination interactions. In addition, the composite membranes also retain intense C─C/C═C peaks, as shown in Fig. 3C, indicating that the sp^2^-hybridized domains remain intact after MOF loading, which is consistent with the Raman spectra (Fig. 3E). This is potentially related to the well-preserved laminar structure stacked by π-π stacking in the fabricated membranes (*25*).

The Cu2p spectra (Fig. 3D) of CuBDC@PGO display two characteristic peaks at 934.5 eV (Cu2p_3/2_) and 953.7 eV (Cu2p_1/2_) (*26*), along with shake-up satellites, confirming that Cu(II) coordination within the layered structured membranes occurs via coordination with carboxyl groups from both the terephthalate (BDC^2−^) and the PGO membrane (*27*). The intensity of the Cu(II) signal in CuBDC@bPGO is approximately one-fourth that of the sPGO-based membrane, which is directly correlated with the oxygen content (table S2). This suggests that CuBDC primarily interacts with PGO via coordination with oxygen functional groups rather than mere physical confinement within interlayer spaces. Theoretically, this coordination occurs through two mechanisms: (i) direct Cu^2+^ coordination with hydroxyl groups, forming metal clusters, and (ii) subsequent interaction of the clusters with carboxyl groups. A slight shift in the Cu(II) peak to a higher binding energy (indicated by arrows in Fig. 3D) supports electron cloud migration from Cu to O atoms, enhancing membrane stability against hydration (*28*, *29*).

The coordination between CuBDC and the sPGO membrane was further corroborated by a G peak shift in the Raman spectrum (Fig. 3E). The G peak shifted from 1598 to ~1608 cm^−1^ (indicated by the green arrow) after confined CuBDC growth in sPGO. Given that the G peak is characteristic of sp^2^-hybridized aromatic carbon arising from the in-plane tangential stretching of C─C bonds, this blue shift likely results from increased bond force constants after p-doping by metal clusters of CuBDC (*30*). Furthermore, the presence of a broad, bump-like 2D peak in all the samples, which corresponds to a second-order Raman process associated with the in-plane breathing modes of the carbon ring, suggests that the overall hexagonal carbon network of the PGO was largely preserved in the composite membranes (*31*).

The x-ray diffraction (XRD) patterns confirmed that structural changes were induced by the incorporation of CuBDC. In Fig. 3F, the peak shifted from 9.83° (bPGO) to 9.56° (sPGO), indicated by the red arrow demonstrating that the d-spacing increased from 8.9 to 9.2 Å. This increase is attributed to enhanced electrostatic repulsion and edge-to-edge interactions among small PGO flakes (*32*, *33*). A distinct peak at ~10.14° in the spectrum of CuBDC@sPGO appeared between the (001) characteristic peak of PGO and the (110) reflection of CuBDC at 10.25°. This suggests that the observed signal results from peak overlap caused by CuBDC coordination as shown in fig. S11. A broad peak at 16.25° to 17.85° corresponds to CuBDC, with a wider full width at half maximum, suggesting a small crystal size according to Scherrer analysis (*34*), which is consistent with confined growth in PGO interlayers confirmed by SEM images. Notably, the (20-1) diffraction peak of CuBDC, which corresponds to crystallographic planes perpendicular to the stacking direction of its layered structure, appears prominently in the XRD pattern. In contrast, bulk characteristic (001) and (020) peaks were absent. This indicates the successful growth of CuBDC nanosheets with preferential orientation aligned along the PGO framework (*27*, *29*, *35*). Compared with the large, randomly oriented CuBDC crystals formed without confinement (figs. S8 and S12), the preferential basal-plane growth of small CuBDC crystallites on PGO is expected to cause only minimal perturbation to the interlayer spacing.

The FTIR spectra shown in fig. S13 further confirmed the formation of CuBDC and its interaction with PGO. The peaks at 1616 and 1422 cm^−1^ correspond to asymmetric and symmetric -COO^−^ stretching, confirming that CuBDC formed via BDC^2−^ coordination with Cu^2+^ (*29*). A −Cu─O stretch at 562 cm^−1^ supported CuBDC incorporation, whereas a new 1116-cm^−1^ C─O─Cu stretch signified coordination with oxygen groups in sPGO (*27*). These interactions enhance interlayer adhesion, bridge voids between adjacent nanosheets, and improve membrane stability (*8*).

**Fig. 4.**
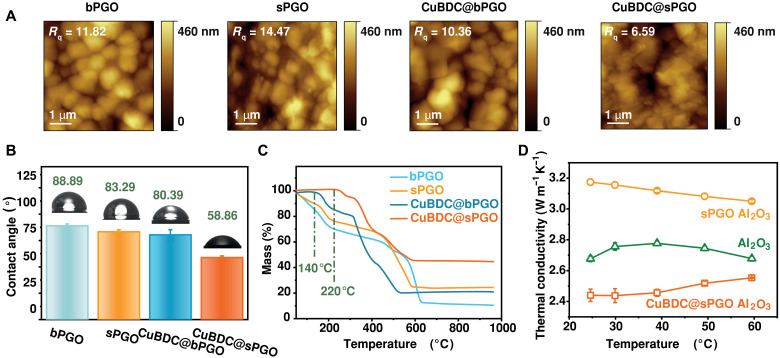
Surface and thermal properties of the membranes. (**A**) Surface roughness measured by AFM. (**B**) Contact angle of the membranes. (**C**) TGA plot of the bPGO, sPGO, CuBDC@bPGO, and CuBDC@sPGO membranes. (**D**) Thermal conductivity of the Al_2_O_3_ HF substrate, sPGO40s HF membrane, and CuBDC@sPGO40s membrane. The error bars indicate the SD of three different measurements.

Surface roughness plays a crucial role in membrane fouling behavior (*36*). AFM analysis (Fig. 4A and fig. S14) revealed a rougher surface for sPGO (*R*_q_ = 14.5 nm) than bPGO (*R*_q_ = 11.8 nm) supported on the alumina substrate. This higher roughness is attributed to the greater density of nanosheet edges in sPGO. Upon CuBDC incorporation, the roughness decreased to 10.4 nm (CuBDC@bPGO) and 6.6 nm (CuBDC@sPGO) because of the smooth morphology of the CuBDC nanosheets. This smoother surface should improve antifouling performance by minimizing contact points for contaminants and reducing pollutant accumulation (*37*).

Surface wettability also plays a decisive role in membrane fouling behavior (*38*). As shown in Fig. 4B, CuBDC@sPGO had a contact angle of 58.86°, indicating enhanced hydrophilicity. This increased water affinity stems from the hydrophilic nature of CuBDC (*39*). In contrast, pristine PGO membranes display a relatively high contact angle (~90°) due to the coexistence of hydrophobic sp^2^ regions and hydrophilic sp^3^ domains (*40*). Improved surface hydrophilicity promotes water adsorption and flux while reducing fouling by forming a protective hydration layer (*41*, *42*).

Figure 4C shows the thermal stability of the membranes. The CuBDC@sPGO composite membrane remained stable up to 220°C, whereas CuBDC@bPGO degraded below 140°C because of its lower coordination density, as evidenced by XPS. Pristine PGO membranes exhibit early weight loss, attributed to the pyrolysis of oxygen-containing functional groups, indicating poor thermal stability (*43*). The improved stability of CuBDC@sPGO arises from the coordination of Cu^2+^ with oxygen functional groups, which strengthens interlayer interactions. The superior stability of CuBDC@sPGO over CuBDC@bPGO highlights the importance of increasing the number of reactive sites for effective stabilization.

Thermal conductivity is a key parameter for membranes used in direct contact membrane distillation (DCMD). Laser flash apparatus measurements (Fig. 4D and table S3) revealed that all the samples had significantly lower thermal conductivities than that of the commercial alumina membranes (18 W m^−1^ K^−1^) (*44*). This highlights the advantageous porous structure of the alumina substrate. The average thermal conductivity of the alumina support was ~2.7 to 2.8 W m^−1^ K^−1^, which increased to 3.0 to 3.2 W m^−1^ K^−1^ after coating the sPGO membrane but decreased to 2.4 to 2.6 W m^−1^ K^−1^ in the case of the CuBDC@sPGO composite membrane. The slight increase after sPGO coating arises from the low-density sp^2^ domains and increased photon scattering (*45*). In contrast, the porous structure of MOFs endows them with ultralow thermal conductivity, which is generally less than 0.5 W m^−1^ K^−1^ (*46*). A membrane with low thermal conductivity offers promising thermal insulation, reducing heat transfer across the membrane and ensuring high thermal efficiency in practical applications such as DCMD for seawater desalination (*47*).

### Desalination performances tested via RO, PV, and WCPV

**Fig. 5.**
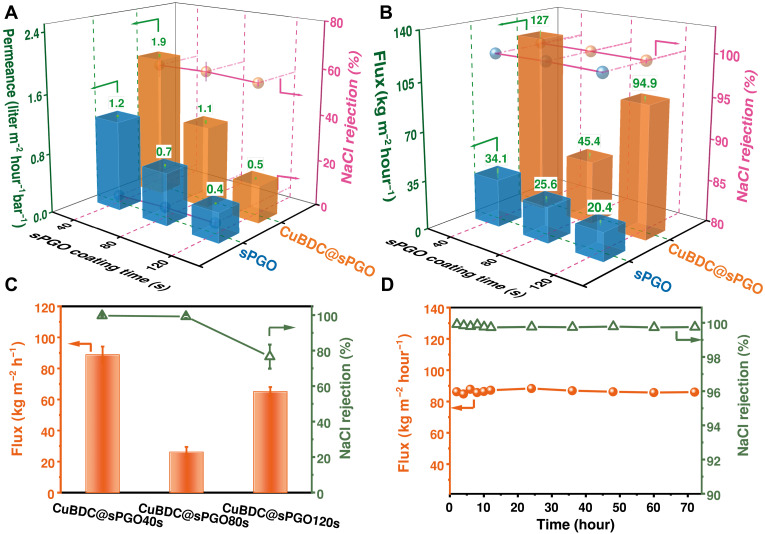
RO and PV performance of the pristine sPGO and CuBDC@sPGO membranes. (**A** to **C**) Flux and NaCl rejection of the sPGO and CuBDC@sPGO membranes tested using the (A) RO system, (B) PV system, and (C) WCPV system. (**D**) Stability test of the CuBDC@sPGO40s composite membrane operated in WCPV mode. The error bars indicate the SD of three different tests.

The desalination performance of the fabricated membranes was evaluated in conventional RO and PV processes using a NaCl feed solution (35 g liter^−1^). The RO performances of sPGO40s, sPGO80s, and sPGO120s before and after CuBDC incorporation are shown in Fig. 5A. As can be seen, increasing the CuBDC loading in the sPGO-based composite membranes significantly enhanced NaCl rejection, increasing from less than 5% to ~55%. This improvement can be attributed to the combined effects of stabilized interlayer spacing and the targeted patching of nonselective defects in the sPGO membrane. Coordination between CuBDC and the functional groups on the sPGO nanosheets effectively suppresses excessive hydration in aqueous environments. Meanwhile, the preferential growth of CuBDC along sheet edges and intrinsic defects selectively patches the originally large, nonselective pores with uniform and well-defined apertures (~5.2 Å), enhancing size-selective transport (*35*). In addition, compared with their pristine sPGO counterparts, the composite membranes exhibited increased permeance. The highest permeance was achieved by CuBDC@sPGO40s at 1.9 liter m^−2^·hour^−1^·bar^−1^, representing a 158% increase over that of the sPGO40s membrane. The improved water permeance is attributed to the increased hydrophilicity imparted by CuBDC, which improves water affinity, facilitates rapid spreading, and promotes water permeance (*48*, *49*). These results demonstrate the successful enhancement of both permeance and salt rejection through confined growth of 2D MOF nanosheets within the sPGO framework. However, the water permeance of CuBDC@sPGO40s was only comparable to that of commercial RO membranes (typically 1 to 2 liter m^−2^ hour^−1^ bar^−1^, e.g., SW30LE58) (*50*).

The composite membranes exhibited excellent desalination performance in conventional PV mode (illustrated in fig. S15), achieving 99.9% salt rejection and a water flux of 124 kg m^−2^·hour^−1^ with CuBDC@sPGO40s, nearly four times greater than that of pristine sPGO40s (Fig. 5B). This exceptional flux surpasses that of most state-of-the-art PV membranes, which typically range from 4 to 80 kg m^−2^ hour^−1^ (*51*, *52*). Similarly, the thicker CuBDC@sPGO80s and CuBDC@sPGO120s composite membranes presented higher water fluxes than their pristine counterparts did, ranging from 35 to 90 kg m^−2^ hour^−1^. The water flux of the sPGO membranes was consistent with the PV performance of previously reported GO membranes (table S4). Compared with bPGO membranes (fig. S16), sPGO membranes exhibited slightly greater flux because of their smaller flakes, which shorten the water transport path (*53*). Notably, the water fluxes of the bPGO and CuBDC@bPGO composite membranes were comparable, largely due to the relatively low CuBDC loading in the CuBDC@bPGO membranes, hence showing negligible effects on the permeation flux.

The reason for two orders of magnitude increases in water flux via PV mode compared to the RO mode is due to differences in the driving forces. During RO operation, i.e., pressure-driven operation, water transport occurs via hydraulic pressure differences, which are relatively low compared with the capillary pressure present in the PV system. Water in the interlayer nanochannels of the PGO membrane does not experience a phase transition in RO mode, leading to slow transport over long and tortuous paths (indicated by the red arrows in Fig. 1D). Conversely, in PV, a liquid-vapor interface forms in the nanochannels, generating high capillary pressure (~1440 bar for GO membranes) (*17*), which drives water efficiently across a large portion of the nanochannel of the PGO membrane’s cross section.

In theory, PV desalination could proceed without a membrane, e.g., via vacuum-assisted evaporation in falling-film evaporators, since salts are nonvolatile (*54*). However, a membrane is introduced to provide a stable vapor(gas)-liquid interface, which is critical for process stability and efficiency under vacuum conditions (*55*). In particular, membranes with a HF geometry can offer surface area–to–volume ratios exceeding 10,000 m^2^/m^3^ (*56*), whereas commercially available falling film evaporators used in vacuum-assisted evaporation have surface area–to–volume ratios of only ~200 m^2^/m^3^. This represents a 50-fold increase in the interfacial area, enabling substantial process intensification (*57*).

The membranes used in these vacuum-driven systems are often nonselective and serve primarily to maintain phase separation rather than to discriminate between permeating species. This configuration is referred to as vacuum membrane distillation (VMD). VMD typically results in higher water flux than PV because of the absence of a selective diffusion barrier. However, PV, which uses selective membranes, offers advantages in terms of high salt rejection and membrane stability, particularly in fouling-prone or complex feedwater environments. These attributes make PV a promising alternative or complement to VMD in challenging seawater desalination scenarios (*58*).

Despite their excellent performance, large-scale PVs are hindered by the high energy demand required to sustain a continuous vacuum on the permeate side (*59*). To address this limitation, a WCPV system (illustrated in fig. S1), which is analogous to the energy-efficient DCMD process, was used to further evaluate the desalination performance of the CuBDC@sPGO40s composite membrane. As shown in Fig. 5C, a high flux of 89 kg m^−2^ hour^−1^ with a promising salt rejection of more than 99.9% was achieved. This flux nearly doubles those of previously reported membranes summarized in table S5, which mostly exhibited fluxes below 50 kg m^−2^ hour^−1^. The slightly lower water flux of 89 kg m^−2^ hour^−1^ compared with 124 kg m^−2^·hour^−1^ obtained via conventional PV mode is due to operational mode differences. Vacuum-driven PVs promote convective vapor flow, whereas WCPVs rely mainly on diffusive transport, resulting in lower flux. In any case, the substantial difference in water flux between the RO and PV modes is due primarily to the stronger driving force in the PV mode, which is absent in the RO mode.

Figure 5C further illustrates that owing to the increased thickness, the flux of the sPGO composite membrane fabricated using sPGO80s was reduced to less than 30 kg m^−2^ hour^−1^. However, the flux of the CuBDC@sPGO120s membrane was nearly twice that of the sPGO80s-based membrane (also observed in conventional PV, as shown in Fig. 5B), but the salt rejection rate decreased to less than 80%. This may be due to the nonuniform distribution of the CuBDC nanosheets within the thicker sPGO membrane, which arises from the different diffusion rates of the metal and ligand solutions, resulting in an imperfect patch of the sPGO membranes. This effect was negligible in thinner sPGO40s matrices. It should be noted that the custom-designed membrane module, as depicted in fig. S1, offers an effective testing area more than 10 times larger than that of the RO and conventional PV systems. This larger testing area demonstrates the scalability potential of sPGO40s composites for desalination.

The PGO membranes patched with CuBDC selectively tested via either conventional PV or WCPV exhibited stable performance over an extended duration. Notably, the CuBDC@sPGO40s membrane, which exhibited the best desalination performance, maintained stable performance over up to a 72-hour continuous WCPV test with both 35,000–parts per million (ppm) (Fig. 5D) and 70,000-ppm NaCl (fig. S17A). These results further demonstrate its robustness and suitability for long-term desalination, including operating under elevated salinity conditions. In contrast, the pristine sPGO40s membrane underwent severe delamination within 1 hour (fig. S17B). The CuBDC@sPGO composite membranes also demonstrated substantial stability in terms of both water flux and NaCl rejection over a 12-hour continuous PV test (fig. S18). Despite low CuBDC loading, CuBDC@bPGO also retained structural integrity over 12 hours of testing. In contrast, the pristine sPGO and bPGO membranes experienced structural failure within 2 to 4 hours (as shown in figs. S18 and S19), showing rising flux but sharp rejection loss. The structural robustness of the composite membrane is attributed to the coordination between the oxygen-containing functional groups on the PGO flakes and the Cu^2+^ clusters in the CuBDC, as confirmed by structural characterization. These interactions mitigate the hydration of oxygen-containing groups, suppressing PGO layer swelling and enhancing membrane integrity under aqueous conditions. This highlights the potential of the CuBDC@sPGO composite membrane for energy-efficient, high-performance PV-based desalination.

### Technoeconomic analysis

**Fig. 6.**
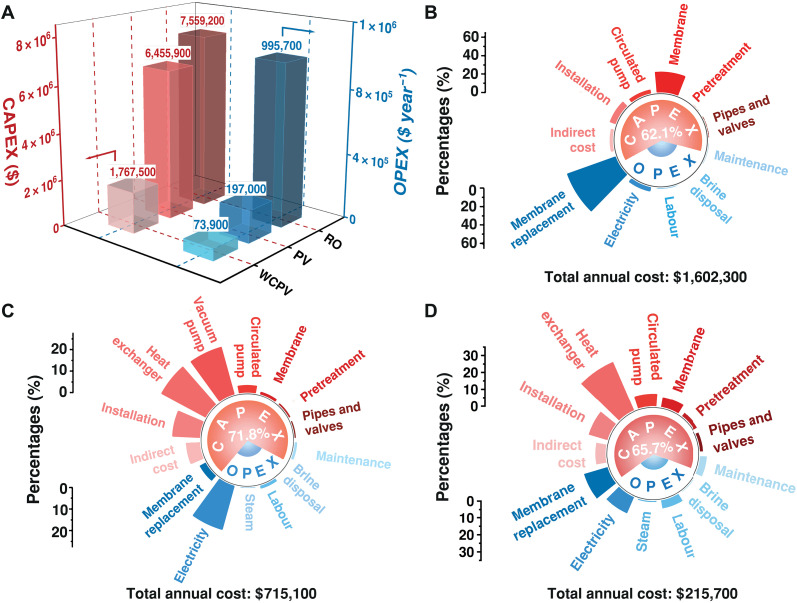
TEA of the CuBDC@sPGO40s composite membrane for desalination. (**A**) Comparison of annual OPEX and total CAPEX operated in the RO, PV, and WCPV modes. (**B** to **D**) Annual cost breakdown by category for plants operated in (B) RO, (C) PV, and (D) WCPV modes.

A comprehensive TEA is essential following the performance assessment of the fabricated membrane (*60*). Here, the economic potential of the CuBDC@sPGO40s HF membrane is operated in three models, RO, PV, and WCPV, for treating saline water (500 m^3^ day^−1^) with total dissolved solids ranging from 35,000 to less than 500 ppm (*61*). The key findings for capital expenditure (CAPEX) and operating expenditure (OPEX), which together characterize the requirements for sustainable deployment (*62*), are presented in Fig. 6. The RO model requires a total capital investment of more than $7.5 million (Fig. 6A). This high CAPEX reflects the large membrane area needed to offset low mass transport efficiency, resulting in substantial membrane procurement and annual replacement costs that dominate the OPEX (Fig. 6B). In contrast, conventional PVs offer higher mass transfer efficiency per unit membrane area, resulting in lower membrane-related costs; however, high capital investment is still needed because of the need for vacuum pumps and heat exchangers. In addition, the electricity required to operate these auxiliary systems accounts for more than 55% of the OPEX of the conventional PV process (Fig. 6C). The WCPV model results in the lowest expenditure among the three models, requiring ~23% RO and 27% PV for capital outlay, whereas its operating expenditure is ~7.4% RO and ~38% PV. When amortized capital charges are combined with annual operating costs (*63*), the total annual cost of WCPV is ~30% that of PV and less than 14% that of RO, justifying the cost efficiency of the CuBDC@sPGO40s membrane in a low-energy process (Fig. 6, B to D). Collectively, these results highlight the strategic value of deploying the CuBDC@sPGO40s composite membrane within low-energy, high-efficiency WCPV architectures to minimize life cycle costs and enable sustainable, large-scale desalination.

## DISCUSSION

In this work, we developed a structurally robust and water-stable CuBDC@PGO composite membrane through the confined in situ growth of 2D CuBDC nanosheets within a PGO framework. The strong coordination between Cu^2+^ ions and oxygen-containing groups on PGO nanosheets effectively mitigates swelling and enhances membrane stability under aqueous conditions. By reducing the flake size of PGO, we increased the density of reactive sites, leading to the increased MOF loading and improved membrane performance.

The resulting membranes demonstrated excellent desalination performance. In RO mode, the water permeance of the composite membrane was comparable to that of current commercial RO membranes. However, when it is operated in conventional PV mode, a record-high flux of 124 kg m^−2^·hour^−1^ with >99.9% salt rejection was achieved, outperforming most existing GO-based and MOF-based membranes. Notably, in a low-energy WCPV process, the membrane maintained a high flux of 89 kg m^−2^·hour^−1^ with excellent long-term stability over 72 hours. The comprehensive TEA analysis further confirmed that WCPV is the most cost-effective mode, offering significantly lower capital and operating costs than both RO and PV.

This study addresses key challenges in developing high-performance MOF/GO composite membranes by achieving a stable laminar structure with improved selectivity at the molecular level. It also introduces a promising strategy for fabricating robust, energy-efficient membranes. These findings open avenues for advanced desalination and other possible separation processes, as the proposed method for fabricating PGO-MOF membranes is versatile and is readily applicable to various PGO-MOF combinations, fully leveraging the chemical diversity of MOFs and their targeted integration into PGO laminae. For example, the in situ growth of hydrophobic MOFs on PGO membranes may find its application in organic solvent nanofiltration. Future studies are expected to extend this approach to other advanced materials, particularly covalent organic frameworks (COFs), to explore its generality and broader application potential. The PGO HF membrane offers a favorable interfacial platform that restricts and controls nucleation and crystal growth, enabling the formation of highly oriented, crystalline, and ultrathin COF layers. These characteristics are likely to benefit high-performance separation applications and warrant further investigation.

## MATERIALS AND METHODS

### PGO nanosheet preparation

Two types of PGO nanosheets are prepared: bPGO and sPGO.

#### bPGO nanosheet preparation

bPGO nanosheets were synthesized as described in our previous work (*19*). Briefly, 80 mg of GO, derived from graphite via a modified Hummer’s method, was dispersed in 20 ml of deionized water under sonication. The resulting dispersion was gently stirred at 50°C for 5 hours following the addition of NH_4_OH (2 ml) and H_2_O_2_ (2 ml). The bPGO nanosheets were then collected by centrifugation at 12,000 rpm for 1 hour at 10°C. To ensure the removal of residual reagents, the collected bPGO was redispersed in deionized water and dialyzed for 3 days.

#### sPGO nanosheet preparation

sPGO nanosheets were synthesized via tip sonication (500 W, 20 kHz; Thermo Fisher Scientific, FB505) under controlled conditions. The PGO dispersion was placed in double-wall glassware, with temperature regulation maintained via a recirculating water chiller. The flake size was precisely tuned by optimizing the sonication parameters, including duration, pulse mode, and amplitude, which were set at 2 hours, 03/01 (on/off), and 30%, respectively.

### PGO HF membrane fabrication

#### Alumina support preparation

The alumina HF substrate was fabricated using a phase inversion method, followed by sintering at 1450°C (*64*). After sintering, the HF substrate, with a diameter of 2.2 mm, was cleaned in a sonication bath with acetone and ethanol before being cut into 4- and 13-cm pieces for subsequent coating. Alumina discs (10-mm diameter by 1.45-mm thickness) were fabricated via the same method for characterization.

#### PGO HF membrane fabrication

PGO membranes were fabricated via a vacuum-assisted self-assembly method. An alumina substrate, with one end sealed by Araldite adhesive and the other connected to a vacuum pump, was immersed in a PGO dispersion (0.025 mg·ml^−1^). The membrane thickness was controlled by varying the coating time to 40, 80, and 120 s, yielding membranes with different thicknesses, labeled PGO40s, PGO80s, and PGO120s, respectively. After coating, the membranes were dried in a vacuum oven at 40°C for 3 hours.

### CuBDC@PGO HF membrane fabrication

#### CuBDC@PGO membrane fabrication

The CuBDC@PGO membrane was synthesized via in situ growth of CuBDC nanosheets within the laminar structure of the PGO membrane via a contradiffusion method. The ligand and metal solutions for CuBDC synthesis were prepared separately. The ligand solution was obtained by dissolving 0.36 g of H_2_BDC in a mixture of 36 ml of *N*,*N*′-dimethylformamide (DMF) and 12 ml of acetonitrile under constant stirring. Similarly, the metal solution was prepared by dissolving 0.36 g of Cu(NO_3_)_2_·3H_2_O in a mixture of 12 ml of DMF and 36 ml of acetonitrile. For the CuBDC patch, the PGO membrane was immersed in the ligand solution (15 ml for the 4-cm HF and 25 ml for the 13-cm HF), while the metal solution was injected into the lumen of the HF. The system was left undisturbed for 24 hours to facilitate CuBDC crystallization. Afterward, the resulting membrane was thoroughly washed in methanol (three cycles of 30 min each) and dried at 40°C overnight. The rationale for selection of important parameters, including reaction time and temperature, has been given in note S2 and fig. S20.

#### *CuBDC@PGO composite membrane fabricated by conventional* in situ *growth*

The same metal salt and ligand solutions used in the counterdiffusion method were premixed before membrane immersion. The coated PGO membranes [sealed at both ends with polytetrafluoroethylene (PTFE) tape] were immersed in the mixed solution in a 15-ml centrifuge tube and reacted at room temperature for 24 hours, followed by washing with methanol (three times) and vacuum drying at 40°C.

### CuBDC HF membrane fabrication

The CuBDC membrane was synthesized on a cleaned alumina HF substrate following the same procedure used for the fabrication of the CuBDC@PGO membrane.

### Characterizations

The sample preparation and instrument details are given in the “Materials and Methods” section in the Supplementary Materials.

### Performance test

#### Desalination performance test via RO

The RO performance of all samples was evaluated via a dead-end filtration system. The prepared HF membrane with effective area ranges from 0.73 to 8.99 m^2^ was mounted within a stainless-steel cylinder containing an NaCl aqueous solution (35 g liter^−1^). The permeate was collected under an applied transmembrane pressure of 10 bar. The permeance ($J_{\text{RO}}$, liter·m^−2^·hour^−1^·bar^−1^) and salt rejection (R, %) were determined via Eqs. 1 and 2, respectively

$$J_{\text{RO}}=\frac{∆w}{∆t\times A\times P}$$ (1)

where ∆w is the weight gain in the permeate, ∆t is the time interval, P is the applied pressure, and A is the effective membrane area

$$R=\left(1-\frac{C_{p}}{C_{f}}\right)\times 100$$ (2)

where $C_{p}$ and $C_{f}$ refer to the NaCl concentrations in the feed and permeate, respectively.

#### Desalination performance test via PV

The PV performance of all samples was evaluated using a custom-built setup (fig. S15). In this setup, the membrane was sealed at one end and connected to a vacuum pump at the other end, providing an effective membrane area of 1.73 cm^2^. A NaCl solution with a concentration of 3.5 weight % (wt %) was circulated over the outer surface of the membrane under continuous stirring at 70°C. A liquid nitrogen cold trap was used downstream between the membrane and the vacuum pump to collect the permeate.

The exact flux J was calculated as follows

$$J=\frac{∆w}{∆t\times A}$$ (3)

where ∆w is the weight gain in the permeate, ∆t is the time interval, and A is the effective membrane area.

The salt rejection was calculated via Eq. 2.

#### *Desalination performance test* via *WCPV*

WCPV was conducted using a laboratory-made setup. As illustrated in fig. S1, an HF module, assembled by potting two pieces of 13-cm HF membranes (with a total effective area of 17.97 cm^2^) with Araldite adhesive, was connected to two flow streams. The feed stream (indicated by the red arrows) containing 3.5 or 7 wt % NaCl circulated over the outer surface of the membrane at a flow rate of 35 ml min^−1^, whereas a cold deionized water stream (blue arrows) continuously circulated through the membrane lumen. Both streams were driven by peristaltic pumps, with the feed stream temperature controlled at 60°C via a hot plate. The pressure on the feed side was regulated to 1 bar via a valve (V). The inlet and outlet temperatures of the feed stream were monitored with thermocouples (*T*_*f1*_, *T*_*f2*_), and the pressure was tracked by pressure sensors (*P*_*f1*_, *P*_*f2*_). Similarly, the permeate side temperatures at the inlet (*T*_*p1*_) and outlet (*T*_*p2*_) were also monitored and recorded. The flow rates on both the feed and permeate sides were measured as *F*_f_ and *F*_p_, respectively. The PV performance was assessed on the basis of the change in weight and conductivity of the permeate tank.

The exact flux J and salt rejection R were calculated by following Eqs. 3 and 2, respectively.

### Technoeconomic analysis

This assessment estimates the total levelized cost of production at the plant scale on the basis of the desalination performance achieved at the bench scale and is expressed in USD per year. Both CAPEX—including costs associated with piping, valves, membranes, pumps, heat exchangers, pretreatment, installation, and related indirect expenses as well as OPEX, covering membrane replacement, electricity, steam, labor, brine disposal, and maintenance—were calculated via eqs. S2 to S35 (*62*). Since the MOF/PGO composite membrane is still at the research stage, no commercial price is available for reference. Therefore, the membrane cost per unit area was estimated as the sum of the material and labor costs, which were calculated from practical experimental data. A summary of all the technical assumptions, total capital costs, annual operating expenditures, and overall annualized costs for each process is provided in the “Define system boundaries and assumptions” section in the Supplementary Materials, along with the detailed data listed in tables S6 to S8.

## Supplementary Materials

### This PDF file includes:

Supplementary Text Figs. S1 to S20 Tables S1 to S8 Notes S1 and S2 References
