# Supplementary material for: Stable porous graphene oxide membranes enabled by confined growth of 2D MOF nanosheets for high-performance desalination
Source: Sci Adv. 2026 May 15;12(20):eaee2550. doi: 10.1126/sciadv.aee2550 (PMC13178534; doi:10.1126/sciadv.aee2550)
Supplement: Supplementary file 1 — Supplementary Text Figs. S1 to S20 Tables S1 to S8 Notes S1 and S2 References [file sciadv.aee2550_sm.pdf]

Science Advances
AAAS

Supplementary Materials for

Stable porous graphene oxide membranes enabled by confined growth of 2D MOF nanosheets for high-performance desalination

Mengjiao Zhai *et al.*

Corresponding author: Kang Li, kang.li@imperial.ac.uk

Sci. Adv. **12**, eaee2550 (2026)
DOI: 10.1126/sciadv.aee2550

This PDF file includes:

Supplementary Text
Figs. S1 to S20
Tables S1 to S8
Notes S1 and S2
References

Supplementary text: Materials and methods

1.1 Materials

All the following chemicals were used without further purification: graphite powder (Sigma Aldrich, 99% carbon basis, 325 mesh), copper nitrate trihydrate ($Cu(NO_3)_2$•$3H_2O$, Sigma Aldrich, 99%), and terephthalic acid (H_2BDC, Sigma Aldrich, 98%). Alumina oxide powder (Inframat Advanced Materials, 99.99%, α-phase, 0.5-1 μm), hydrogen peroxide (H_2O_2, Merck, 30%), ammonium hydroxide (NH_4OH, 28--30%), ethanol (VWR, 99.5%), acetone (VWR, 99.5%), anhydrous N, N-dimethylformamide (DMF, VWR, 99%), acetonitrile (CH_3CN, VWR, 99.9%), chloroform ($CHCl_3$, VWR, 99.9%), methanol (VWR, 98.5%), and sodium chloride (NaCl, VWR, 98%) were used.

1.2 Synthesized CuBDC crystals using the bottom-up method

CuBDC crystals have been synthesized using a reported bottom-up method (9). Briefly, a three-layer solution system was constructed in a 1 L Duran® bottle. The bottom layer consisted of a linker solution containing 1.8 g H_2BDC dissolved in a mixed solvent of 240 mL DMF and 120 mL CH_3CN. The middle layer comprised a solvent mixture of 120 mL DMF and 120 mL CH_3CN. The top layer was a metal precursor solution containing 1.8 g $Cu(NO_3)_2 \cdot 3H_2O$ dissolved in 120 mL DMF and 240 mL CH_3CN. The middle and top layers were carefully added dropwise to prevent disturbance of the underlying solution. The resulting three-layer system was maintained statically at room temperature for 24 h to allow interfacial growth. The obtained products were subsequently collected by centrifugation, washed three times each with DMF and $CHCl_3$, and finally dispersed in CH_2Cl_2 for characterization.

1.3 Characterizations

The surface and cross-sectional morphologies of the membranes were observed by scanning electron microscopy (SEM, LEO 1525, Zeiss) with a 15 nm gold sputter coating to increase the surface conductivity and improve image quality. The elemental distributions of carbon, copper, and aluminium were detected via energy-dispersive X-ray spectroscopy coupled with SEM (SEM-EDX, LEO 1525, Zeiss). The flake size of the nanosheets and surface roughness of the membranes were measured using atomic force microscopy (AFM, MFP-3D, Oxford Instrument). The flake size distribution was also evaluated via dynamic light scattering (Litesizer DLS, Anton Paar). X-ray photoelectron spectroscopy (XPS, K-Alpha$^+$, Thermo

Scientific) was used to measure the surface chemistry of the membranes. The functional groups of the membranes were detected via Fourier transform infrared (FTIR) spectroscopy (Spectra 100, PerkinElmer) over 400–3600 nm at a resolution of 4 cm^{-1}. X-ray diffraction (XRD) analysis was conducted with an X'Pert PANalytical instrument operating over a 2θ range of 2.5° to 20° with 40 kV and a current of 20 mA. Thermal gravimetry analysis (TGA, TGA-8000, PerkinElmer) was conducted to evaluate the thermal stability of the membrane in the temperature range of 50–1000 °C at a heating rate of 10 °C min^{-1} in a N_2 atmosphere. Raman characterization was conducted with a SENTERRA II instrument (Bruke). The wettability of the membrane was examined via water contact angle analysis (Ramé-hart Model 590, Advanced Automated Goniometer).

The thermal conductivity (K, W m^{-1} K^{-1}) of the membranes was evaluated via the following equation (S1):

$$K = D \times \rho \times C_p \quad \text{Equation S1}$$

where D refers to the thermal diffusivity measured via a laser flash apparatus (LFA, NETZSCH) over a temperature range from room temperature to 60 °C. The heating rate was 1 °C per minute, and three measurements were recorded every 10 minutes. ρ is the density of the samples, which is determined based on their dimensions and weight. The specific heat capacity C_p was measured using a Pyris-1 differential scanning calorimeter (DSC, PerkinElmer) according to the method described in the ASTM E1269−11 standard.

Supplementary Figures

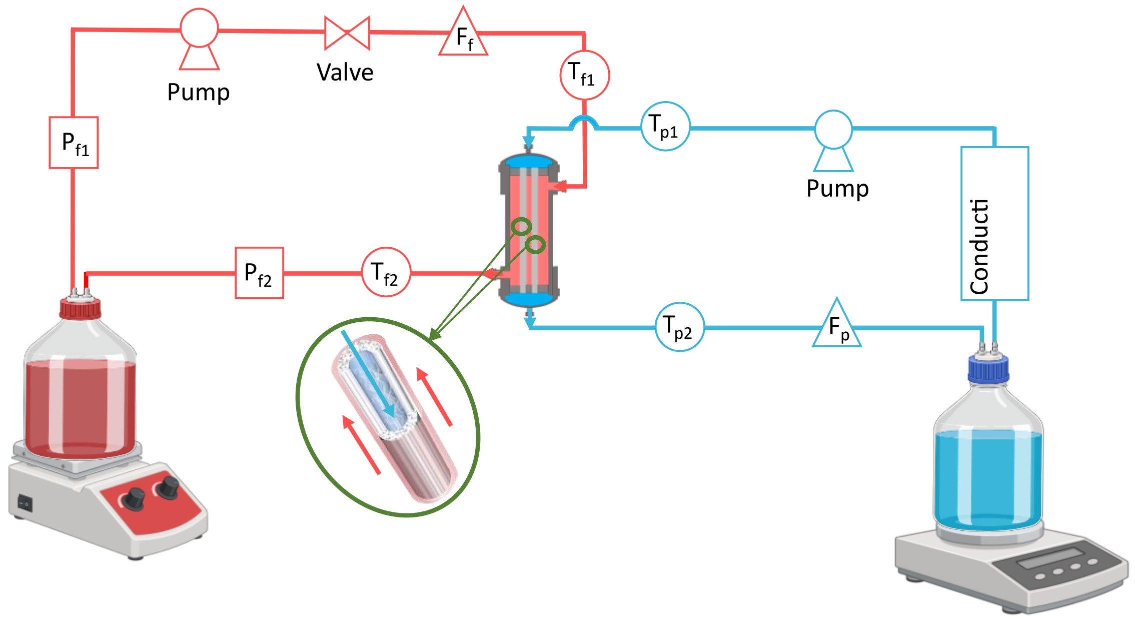

Fig. S1.

Illustration of water carrier pervaporation (analogous to direct contact membrane distillation (DCMD). The membrane module contains two pieces of HF membranes, providing an effective test area of 17.97 cm^2.

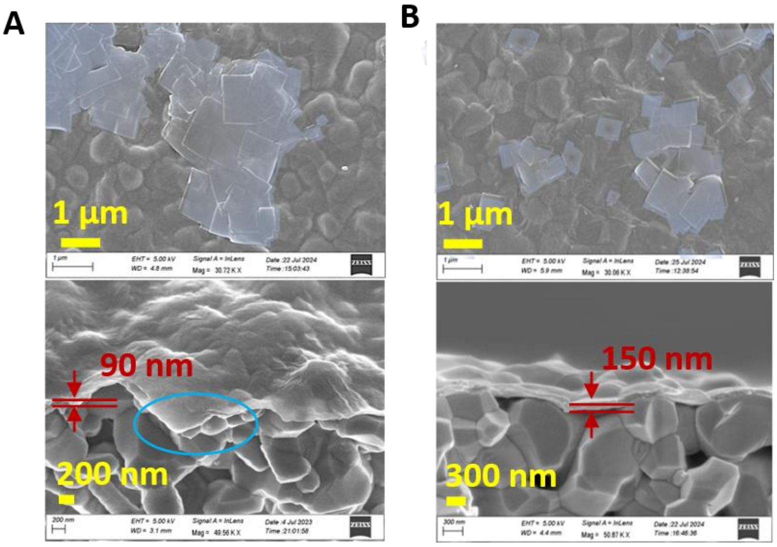

Fig. S2.

Surface and cross-sectional morphologies of (A) CuBDC@bPGO80s, and (B) CuBDC@bPGO120s. The CuBDC nanosheets in a, and b are highlighted in light blue for clarity, and blue circle in (A) indicates the square-edged CuBDC nanosheets.

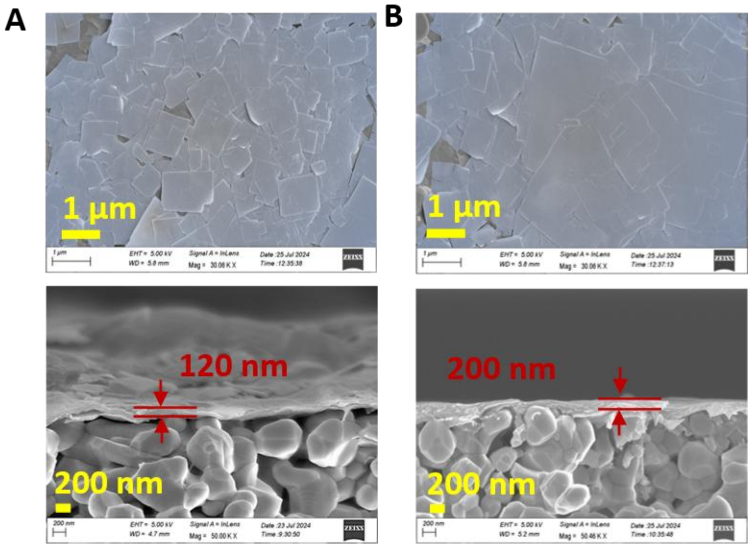

Fig. S3.

Surface and cross-sectional morphologies of (A) CuBDC@sPGO80s, and (B) CuBDC@sPGO120s. The CuBDC nanosheets in a, and b are highlighted in light blue for clarity.

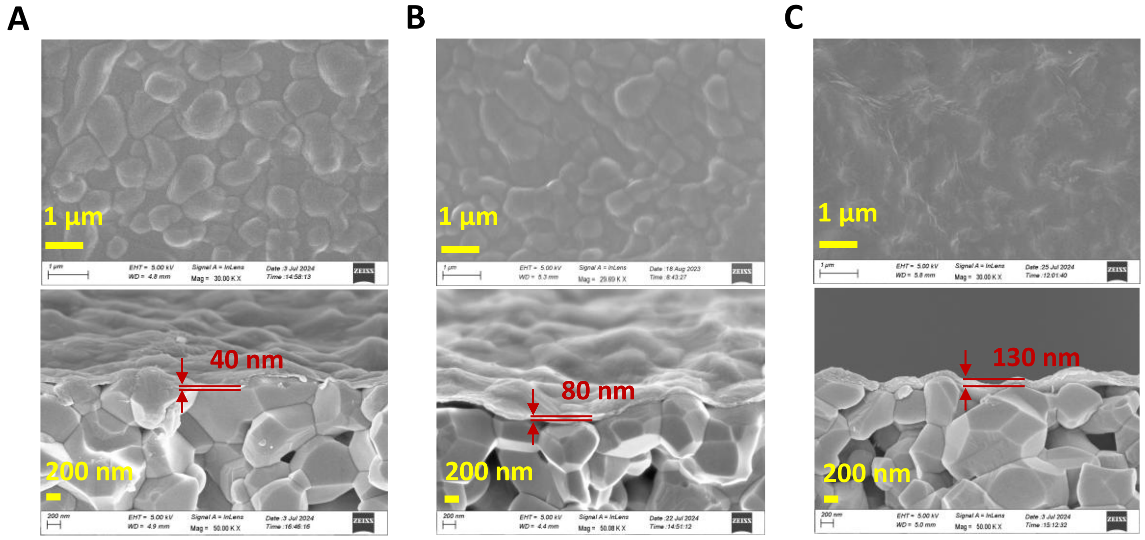

Fig. S4.

Surface and cross-sectional SEM images of (A) bPGO40s, (B) bPGO80s, and (C) bPGO120s**.**

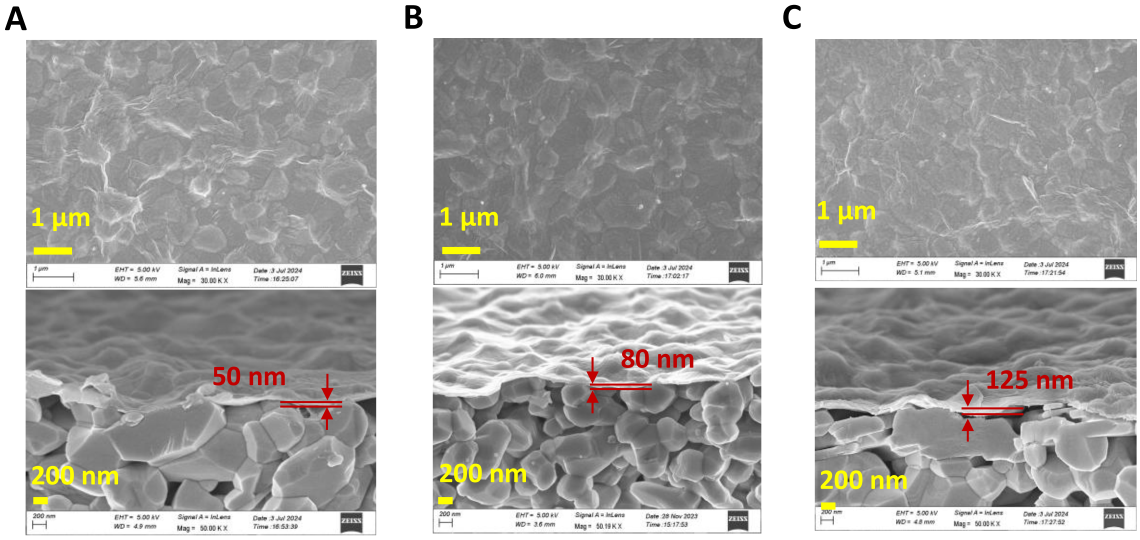

Fig. S5.
Surface and cross-sectional SEM images of (A) sPGO40s, (B) sPGO80s, and (C) sPGO120s.

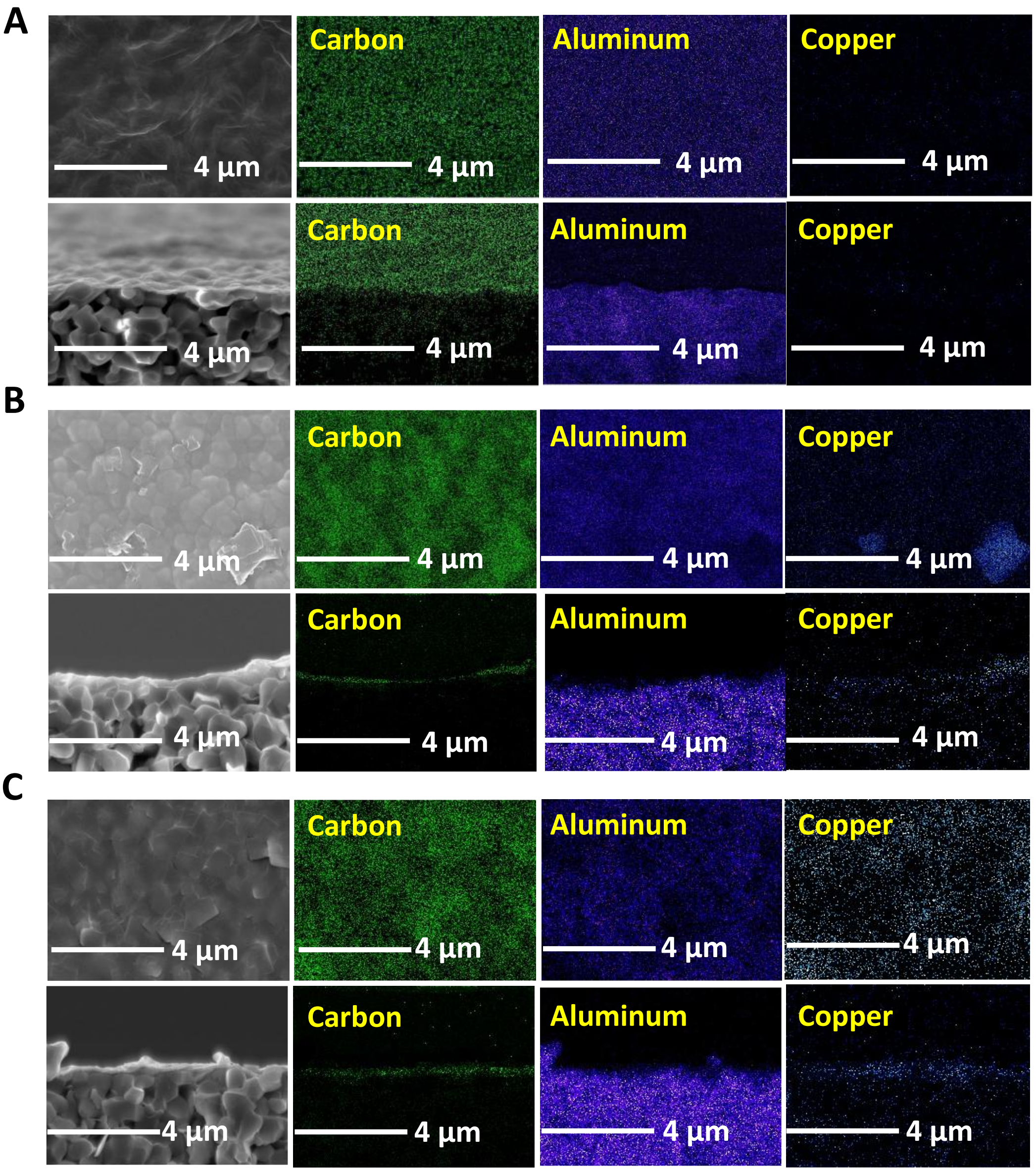

Fig. S6.

Surface and cross-sectional EDX mapping analysis of (A) bPGO40s, (B) CuBDC@bPGO40s and （C) CuBDC@sPGO40s membranes.

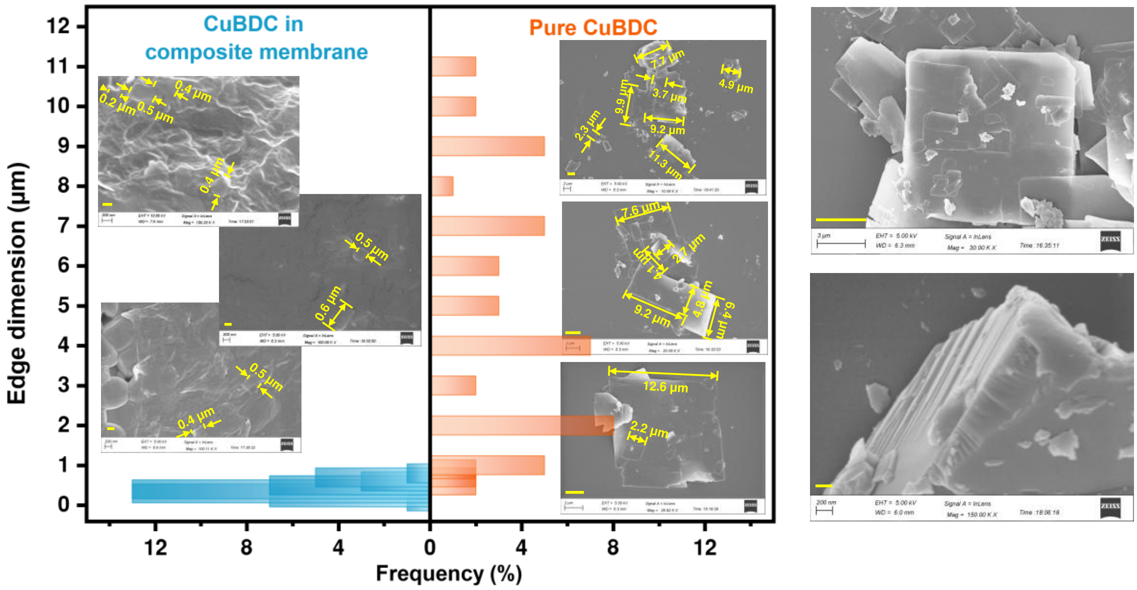

Fig. S7.

Comparison of CuBDC in composite membrane and pure CuBDC**.** (A) SEM images of CuBDC nanosheets in the exfoliated composite membrane and of pure CuBDC. The lateral dimensions were measured using ImageJ, and the size distribution was determined from 50 nanosheets randomly selected from the SEM images. (B) XRD patterns of pure CuBDC and the CuBDC@sPGO composite membrane.

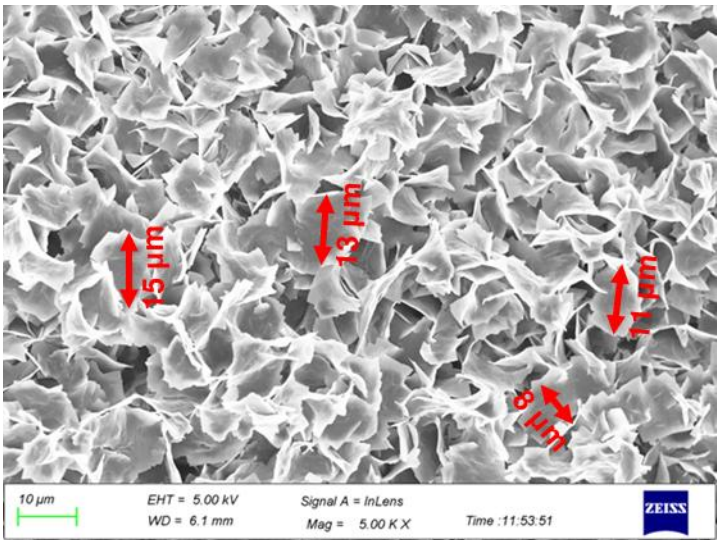

Fig. S8.
SEM of the CuBDC layers fabricated on bare alumina HF

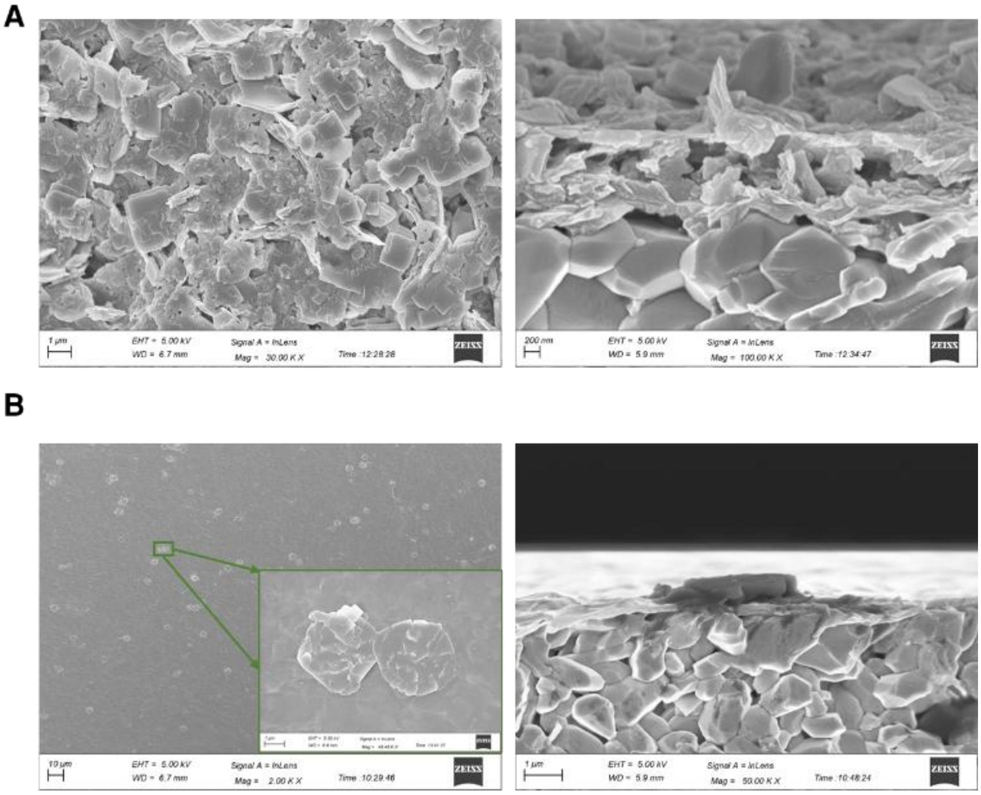

Fig. S9.

Morphology of CuBDC@PGO composite membrane fabricated by vacuum coating-growth and conventional in situ growth method. (A) Surface and cross-sectional SEM images of the CuBDC@sPGO composite membrane fabricated via vacuum-assisted self-assembly. (B) Surface and cross-sectional SEM images of the CuBDC@sPGO40s membrane prepared using the conventional in situ growth method. The green arrows indicated the enlarged view of the selected area.

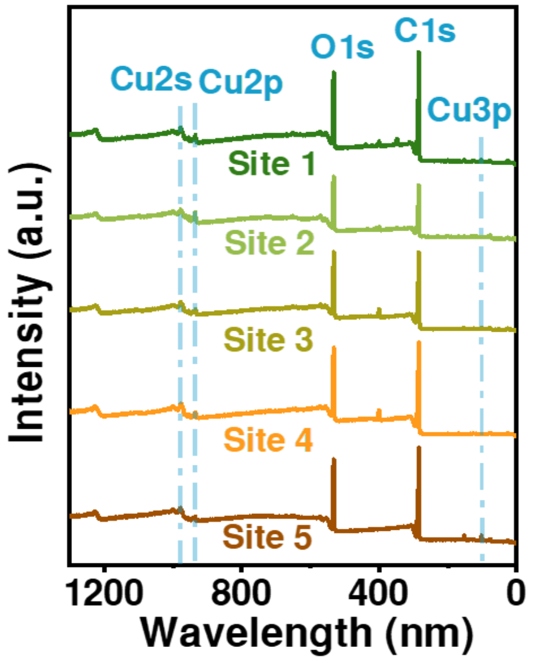

Fig. S10.

XPS spectra of the exfoliated CuBDC@sPGO40s membrane; spectra were collected from randomly selected sites on different exfoliated layers.

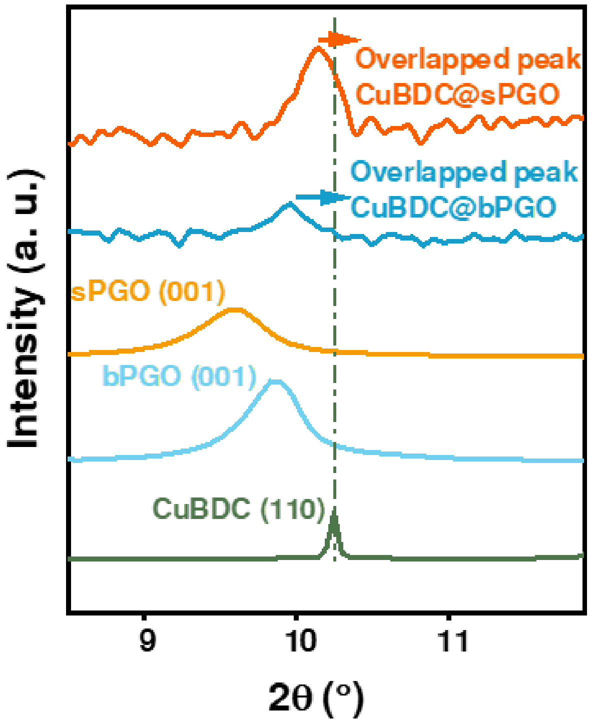

Fig. S11.

Enlarged XRD patterns in the 2θ range of 8.5°–12° for CuBDC, bPGO, sPGO, CuBDC@bPGO, and CuBDC@sPGO samples.

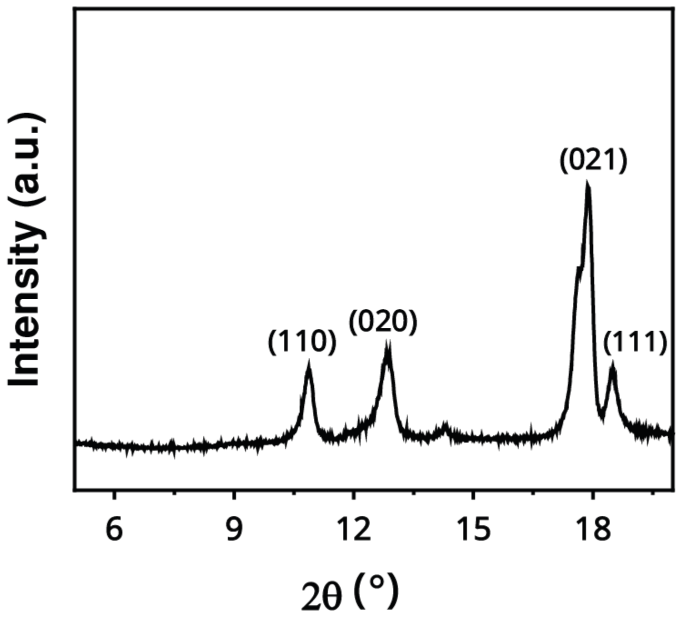

Fig. S12.
XRD of the CuBDC layers fabricated on bare alumina HF.

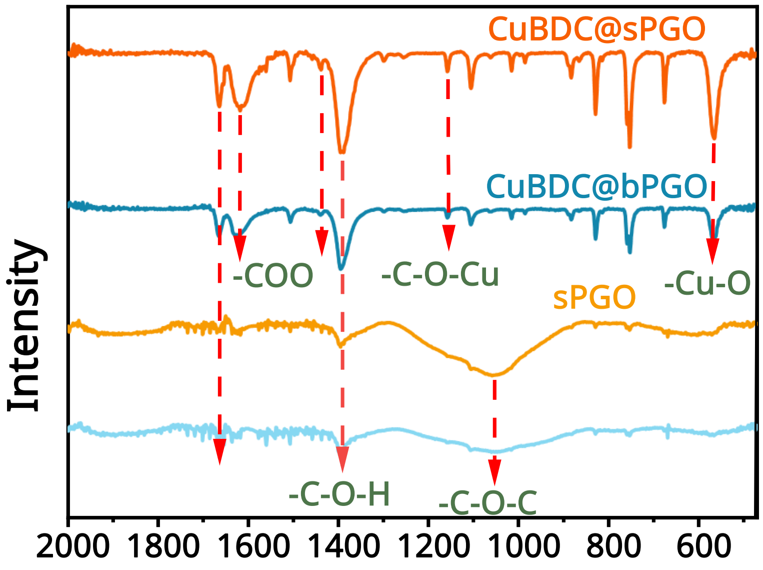

Fig. S13.

FTIR spectra of the bPGO40s, sPGO40s, CuBDC@bPGO40s and CuBDC@sPGO40s membranes.

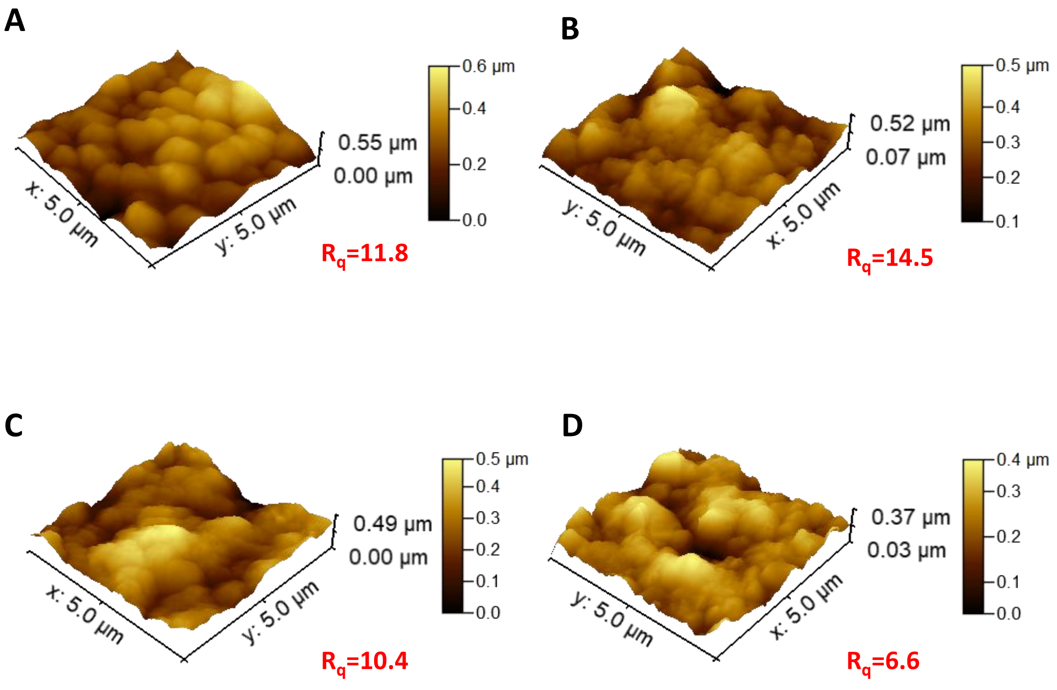

Fig. S14.

3D AFM images of (A) bPGO40s, (B) sPGO40s, (C) CuBDC@bPGO40s, and (D) CuBDC@sPGO40s membranes.

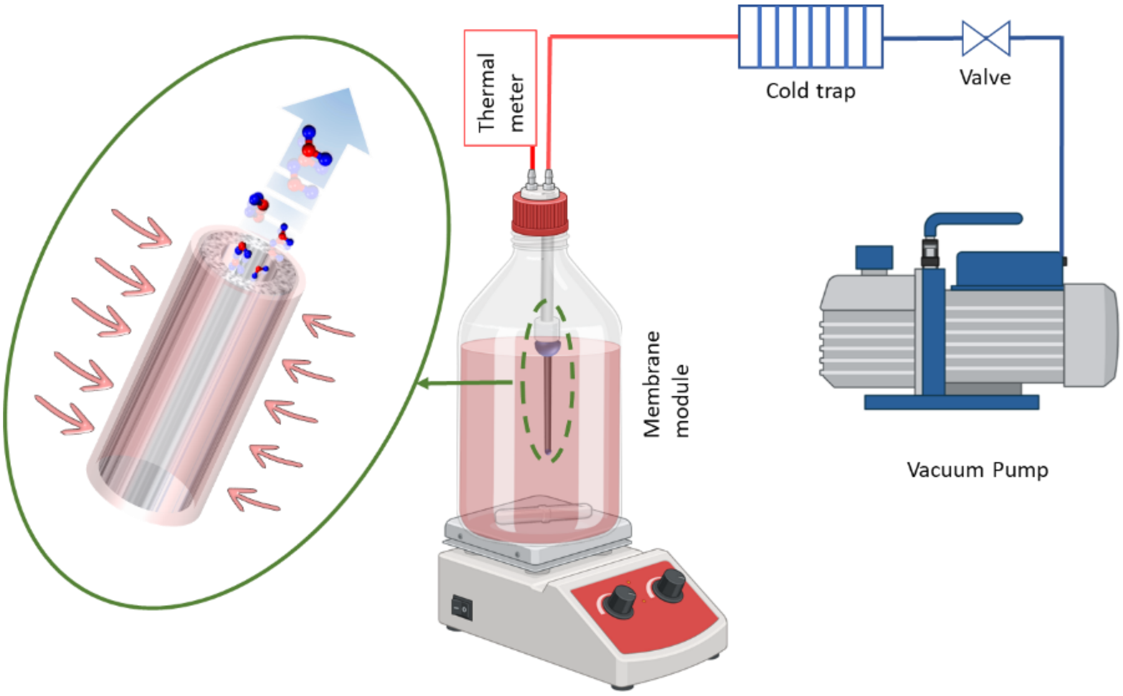

Fig. S15.
Illustration of the pervaporation setup. The effective membrane area is approximately 1.73 cm^2.

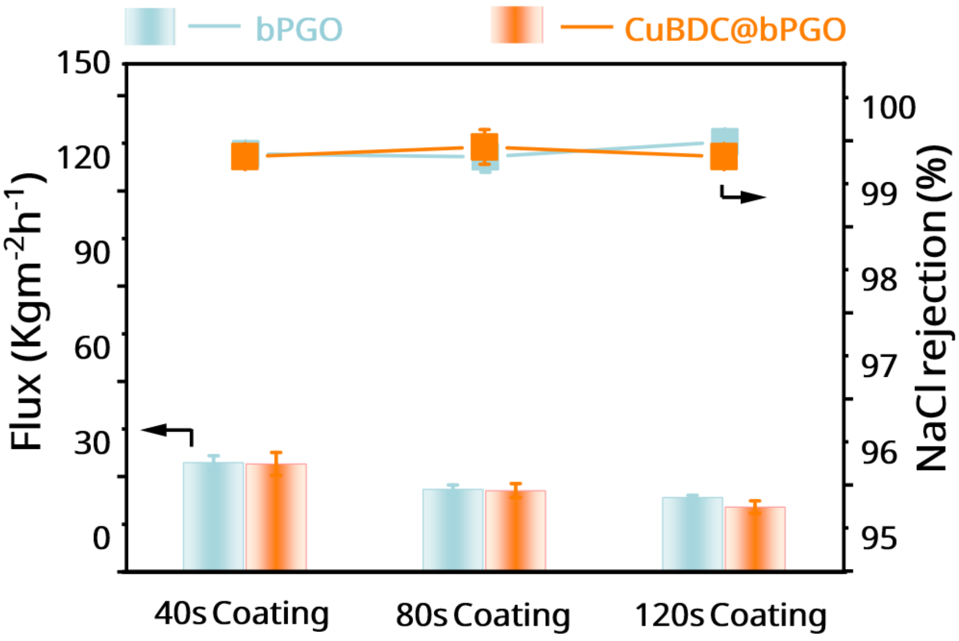

Fig. S16.

Flux and NaCl rejection of the bPGO and CuBDC@bPGO membranes as determined via pervaporation. The error bars indicate the standard deviation of three different measurements.

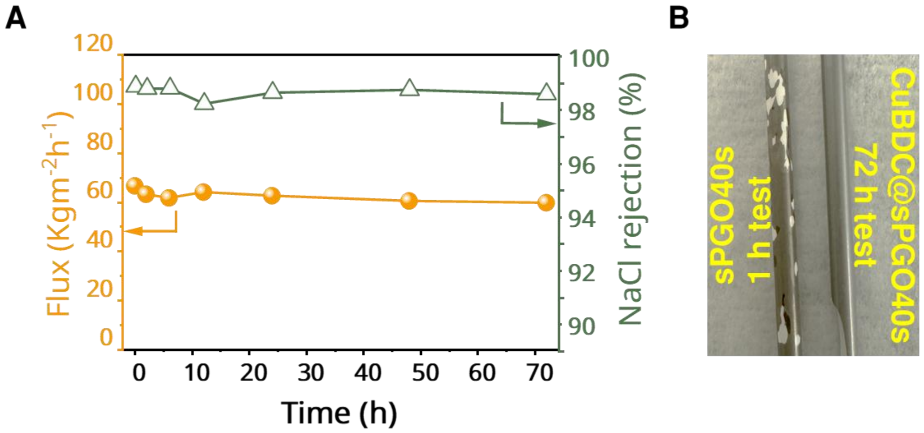

Fig. S17.

Stability test of membranes using 70,000 ppm NaCl as feed. (A) Long-term desalination stability of the CuBDC@sPGO40s membrane during WCPV operation with 70,000 ppm NaCl feed water. (B) Photographs of the pristine sPGO40s membrane after 1 h of WCPV and the CuBDC@sPGO40s membrane after 72 h of WCPV test.

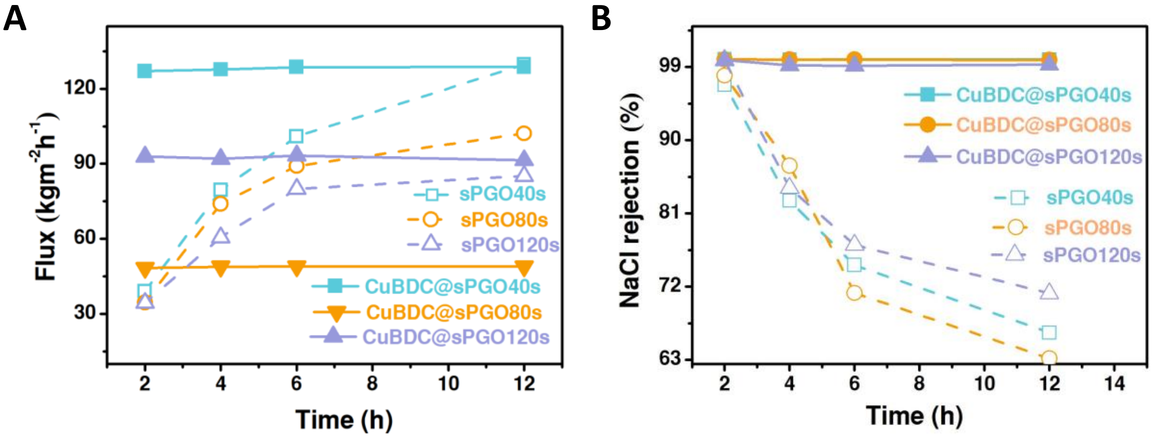

Fig. S18.

(A) Flux and (B) NaCl rejection performance of the sPGO and CuBDC@sPGO composite membranes in the PV test as a function of time.

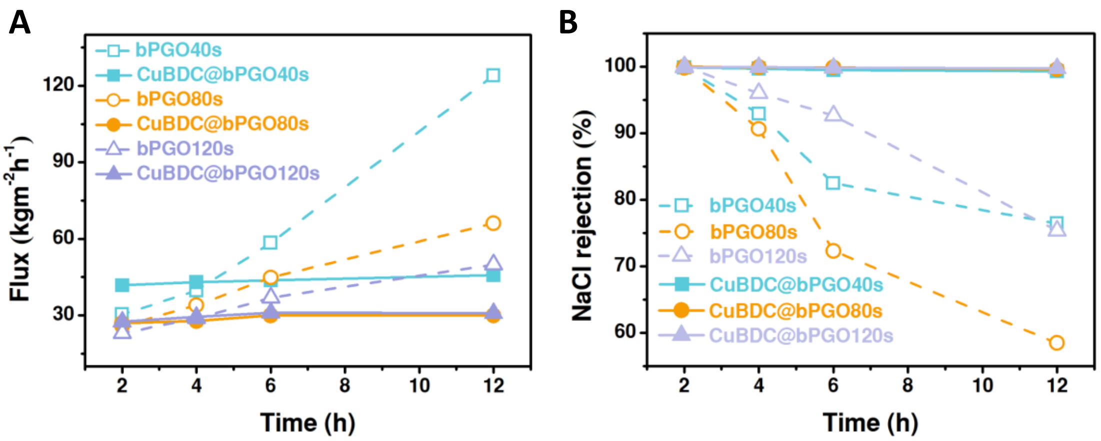

Fig. S19.

(A)Flux and (B) NaCl rejection (B) performance of bPGO and CuBDC@bPGO composite membranes in the PV test as a function of time.

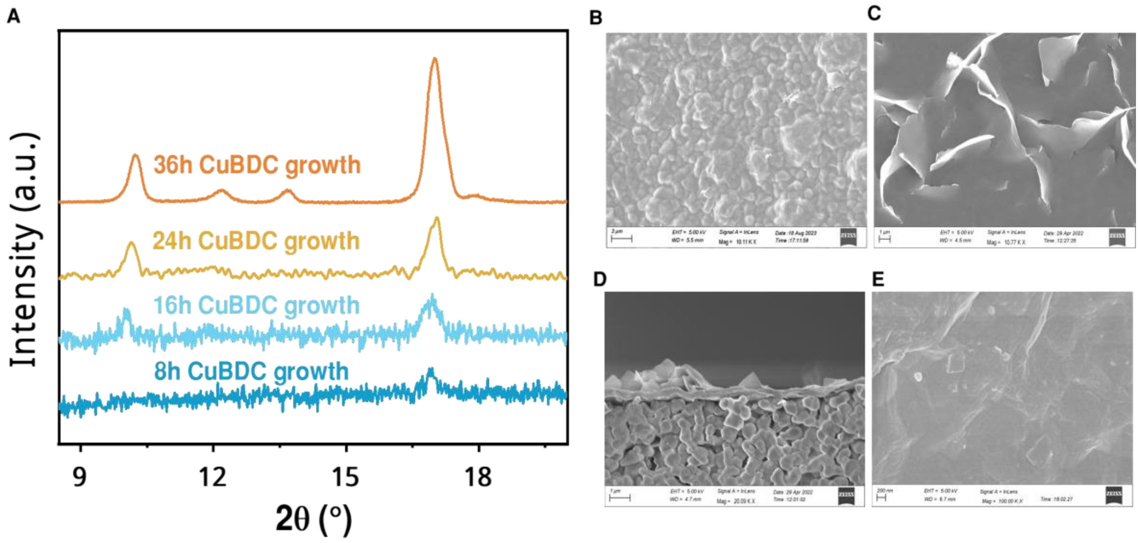

Fig. S20.

XRD patterns and SEM images of CuBDC@sPGO40s membranes fabricated with different MOF growth durations**.** (A) XRD spectra of CuBDC@sPGO40s composite membrane synthesized at various CuBDC growth times. (B) Surface SEM of the CuBDC@sPGO composite memb rane with 8 h MOF growth. (C) Surface SEM of the CuBDC@sPGO composite membrane with 36 h MOF growth. (D) Cross-sectional SEM of the CuBDC@sPGO composite membrane with 36 h MOF growth. (E) Morphology of the 36 h-grown membrane retained on the HF surface after mechanical exfoliation.

Supplementary tables

Table S1 Comparison of membrane thickness before and after the growth of CuBDC

Membrane	Thickness (nm)	Increase ratio (%)
bPGO40s	40	/
CuBDC@bPGO40s	45	13
bPGO80s	80	/
CuBDC@bPGO80s	90	12
bPGO120s	130	/
CuBDC@bPGO120s	150	15
sPGO40s	50	/
CuBDC@sPGO40s	80	60
sPGO80s	80	/
CuBDC@sPGO80s	120	50
sPGO120s	125	/
CuBDC@sPGO120s	200	60

Table S2 Atomic percentages of C, O, and Cu in the fabricated membranes.

Membrane	Carbon (Atomic percentage, at%)	**Oxygen (Atomic percentage, at%)**	**Copper (Atomic percentage, at%)**
bPGO	75.5	25.4	/
sPGO	70.6	29.4	/
CuBDC@bPGO	67.2	25.4	**5.0**
CuBDC@sPGO	38.4	36.7	**21.5**

Table S3 Parameters used for calculating the thermal conductivity of the fabricated membranes

Samples	Temperature (°C)	ρ (g m^3)	α (m^2 s^{-1})	C_p (J g^{-1} K^{-1})	K (W m^{-1} K^{-1})
	24.6	1.108x10 ^{6}	2.780x10^{-9}	0.870	2.68
	29.9	1.108x10 ^{6}	2.769x10^{-9}	0.898	2.76
Al_2O_3	39.0	1.108x10 ^{6}	2.671x10^{-9}	0.938	2.78
	49.3	1.108x10 ^{6}	2.543x10^{-9}	0.974	2.75
	59.5	1.108x10 ^{6}	2.416x10^{-9}	1.000	2.68
	25.4	1.108x10 ^{6}	2.975x10^{-9}	0.918	3.17
	30.0	1.108x10 ^{6}	2.889x10^{-9}	0.940	3.16
sPGO Al_2O_3	39.2	1.162x10 ^{6}	2.730x10^{-9}	0.983	3.12
	49.6	1.162x10 ^{6}	2.572x10^{-9}	1.031	3.08
	58.6	1.162x10 ^{6}	2.455x10^{-9}	1.069	3.05
	25.9	9.485x10 ^{5}	2.852x10^{-9}	0.902	2.44
CuBDC	29.9	9.485x10 ^{5}	2.728x10^{-9}	0.942	2.44
@sPGO	39.3	9.485x10 ^{5}	2.610x10^{-9}	0.992	2.46
Al_2O_3	49.5	9.485x10 ^{5}	2.481x10^{-9}	1.070	2.52
	59.0	9.485x10 ^{5}	2.393x10^{-9}	1.125	2.55

Table S4 Comparison of state-of-the-art desalination membranes via PV systems

Membrane	Substrate	Feed	Temperature (K)	Flux ($Kgm^{-1}h^{-1}$)	Salt rejection (%)	Ref.
**CuBDC@sPGO40s**	**Alumina HF**	**35,000 ppm NaCl**	**343**	**127±1.29**	**99.93**	**This study**
GO-PVA	Cellulose	200,000 ppm NaCl	358	98.1	99.99	(*65*)
GO-PI	/	35,000 ppm NaCl	363	15.6	99.80	(*66*)
GO	PAN	35,000 ppm NaCl	363	65.1	99.80	(*67*)
GO	PAN	35,000 ppm	303	14.3	99.80	(*67*)
GO	PTFE	35,000 ppm NaCl	343	124	99.99	(*68*)
1,4-Diaminobutane-GO	α-Al2O3	Simulated seawater	363	19.7	99.9	(*69*)
GO-Polyimide	/	Simulated seawater	363	36.1	99.9	(*70*)
GO-Polyimide	/	Simulated seawater	363	15.6	99.8	(*66*)
GO-Polyamide	PAN	Simulated seawater	343	26.7	99.99	(*71*)
GO-Chitosan	/	50,000 ppm NaCl	354	30	99.99	(*72*)
GO-SA	/	Simulated seawater	333	8.1	99.41	(*73*)
GO-PEI	/	200,000 ppm NaCl	338	8.4	99.90	(*74*)

GO-PEI	MCE	100,000 ppm NaCl	363	86	99.99	(*75*)
GO@CuBTC/PVDF	/	Simulated seawater	348	36.8	99.68	(*76*)
PA-mCNT	PAN	Simulated seawater	343	104	99.99	(*77*)
MXene	PAN	35,000 ppm NaCl	338	85.4	99.5	(*78*)
MOF-303/MXene-PVA	PAN	35,000 ppm NaCl	308	15.9	99.84	(*79*)
MIL-101(Cr)@GO-PVA	/	40,000 ppm NaCL	333	9.7	99.9	(*80*)
GO-g-PVA	PAN	35,000 ppm NaCl	308	4.5	99.99	(*81*)
PVA/NC	PAN	200,000 ppm NaCl	343	103.1	99.8	(*82*)
BTCA-TAPB-COF	Alumina	35,000 ppm NaCl	353	40	99.9	(*83*)

Table S5 Comparison of WCPV membranes with state-of-the-art desalination membranes via direct contact membrane distillation

Membrane	Substrate	Feed	Temperature difference (ΔK)	Flux ($Kgm^{-1}h^{-1}$)	Salt rejection (%)	Ref.
**CuBDC@sPGO40s**	**Alumina HF**	**35,000 ppm NaCl**	**50**	**88.9±5.21**	**99.70**	**This study**
GO-PVA/SSA	PP	35,000 ppm NaCL	40	3.4	99.60	(84)
GO/PVA& PP-PVDF	/	/	40	52	~100.00	(85)
PET	/	75,000 ppm NaCl	50	1.9	91.39	(86)
Silica-PDA-PVDF	/	35,000 ppm NaCl	50	37.0	~100	(87)
SiNPs/PFOTS	/	250,000 ppm NaCl	40	17.5	~100	(88)
PDA-PEI	PVDF	40,000 ppm NaCl	40	15	99.99	(89)
MOF-808/PVDF/PAN	/	35,000 ppm NaCl	30	4.4	99.97	(90)
UiO-66-NH_2/PVA	PTFE	35,000 ppm NaCl	50	21.3	99.99	(91)
Silica-CA	PTFE	24,000 ppm NaCl	33	20	~100.00	(92)
CNT -PVDF	/	30,000 ppm NaCl	60	74.7	99.99	(93)
PVDF	/	35,000 ppm	40	38.0	99.99	(94)

		NaCl				
ZnO/PVDF	/	Simulated seawater	40	15.7	99.90	(95)
PLA@SiO_2	/	35,000 ppm NaCl	40	14.3	99.99	(96)
12LF-NC	Nylon/nitro cellulose	100,000 ppm NaCl	40	10.1	~100	(97)
9LF-NC	Nylon/nitro cellulose	35,000 ppm NaCl	40	14.0	~100	(97)
Vinyl-POSS-PTFE	/	35,000 ppm NaCl	40	40	99.99	(98)
AlFu-PVA-SSA	PTFE	35,000 ppm NaCl	~35	45.1	99.9	(99)

Abbreviations used in tables S4 and S5:

PVA: Polyvinyl alcohol

PAN: Polyacrylonitrile

PTFE: Polytetrafluoroethylene

PVDF: Polyvinylidene fluoride

PI: Polyimide

PA: Polyamide

SA: Sodium alginate

CA: Cellulose acetate

PEI: Polyethyleneimine

MOF: Metal organic frameworks

MCE: Mixed cellulose ester

CNT: Carbon nanotube

BTCA: 1,3,5-triformylbenzene

TAPB: 1,3,5-Tris(4-aminophenyl)benzene

COF: Covalent organic frameworks

PDA: Polydopamine

PP: Polypropylene

SSA: Sulfosuccinic acid

DADD: 1,12-diaminododecane

PET: Poly(ethylene terephthalate)

PLA: Polylactic acid

HFP: Cohexafluoropropylene

LF: LUMIFLON™ LF-200

NC: Desmodur® N3300

AlFu MOF: Aluminum fumarate metal organic framework

SSA: Sulfosuccinic acid

Supplementary Note 1: Techno-economic analysis (TEA)

This assessment focuses on estimating the total levelized cost of production at the plant scale, expressed in USD per year, comprising both capital expenditure (CAPEX) and operating expenditure (OPEX), representing the full investment required for sustainable operation (*62*).

4.1 Define System Boundaries and Assumptions

The processes are designed for a treatment capacity of 500 m³ of seawater per day, operating continuously for 24 hours a day, 365 days a year. The treatment objective is to reduce the total dissolved solids (TDS) concentration in the feedwater from an initial value of 35,000 ppm to below 500 ppm, in compliance with the recommended health advisory standards(*61*). Three independent desalination plants are developed based on distinct technologies: RO, PV, and WCPV. Each system is engineered to operate continuously 24 hours per day to ensure consistent water production and meet the specified treatment capacity.

4.2 Technical Data

The key technical data are listed in Table S6.

Table S6 Technical data of the RO, PV and WCPV processes

	Salt rejection (%)	Flux	Membrane area (m^2)	TDS in permeate (ppm)
RO[1]	56.5	18 L m^{-2} h^{-1} (10 bar)	6,943.8	237
PV	99.9	118 kg m^{-2} h^{-1}	176.6	35
WCPV	99.7	87 kg m^{-2} h^{-1}	239.5	105

[1] Based on the rejection measured in the reverse osmosis, 6 nanofiltration steps are required to reach the desired TDS.

[2] The required membrane area (Am, m^2) is calculated by Equation S2:

$$Am = \frac{F \times 1000}{t_w \times J} \quad \text{Equation S2}$$

where F (m^3 day^{-1}) is the plant capacity, J (kg h^{-1}) refers to the flux through the membrane, and t_w is the number of working hours per day.

4.3 CAPEX

CAPEX refers to the direct (D_c) and indirect capital costs (ID_c). The direct cost, covering cost of pretreatment (C_{pt}), membrane cost (C_m), equipment acquisition and installation (C_{ins}).

4.3.1 Pretreatment, C_{pt}

The pretreatment process was introduced prior to the desalination process to reduce general aging and destruction of the membrane by suspended matter, oil, iron and microbiological contaminants(*100*). The capital cost of pretreatment (C_{pt}, \$) can be calculated based on the plant capacity (F, m^3 day^{-1}) and pretreatment coefficient (k_{pt}, \$ m^{-3} day^{-1}) adjusted with the most recent chemical engineering plant cost indices (CEPCIs) for estimating the updated price(*101*).

$$C_{pt} = k_{pt} \times F \times \frac{CEPCI_{2024}}{CEPCI_{2009}} \qquad \text{Equation S3}$$

4.3.2 Pipes and valves, C_{pv}

Pipes function as conduits that interconnect various system components, ensuring appropriate flow rates and minimizing pressure losses. Valves regulate the flow direction, isolate specific sections for maintenance, and control operating pressures to prevent membrane damage. From an economic perspective, the cost of pipes and valves (C_{pv}, \$) is closely correlated with the total installed membrane area (Am, m^2), as larger membrane systems require higher flow capacities, longer piping lengths, and a greater number of valves for effective flow management and isolation. The cost of pipes and valves is calculated using Equation S4.

$$C_{pv} = k_{pv} \times Am \times \frac{CEPCI_{2024}}{CEPCI_{2000}} \qquad \text{Equation S4}$$

where k_{pv} is the cost coefficient of pipes and valves suggested in the Sethi & Wiesner cost model published in 2000 (*102*).

4.3.3 Membrane cost, C_m

The total membrane cost (C_m, \$) is determined via Equation S5, where the membrane cost per unit area (C_m, \$ m^{-2}) is the sum of the material cost (C_{mm}, \$ m^{-2}) and labour cost (C_{ml}, \$ m^{-2}). The detailed breakdown of the material costs is provided in Table S7.

$$C_m = Am \times Cm \qquad \text{Equation S5}$$

Table S7 Summary of the material cost for membrane fabrication

Materials	Amount (g m^{-2})	Unit cost (\$ g^{-1})	Cost (\$ m^{-2})
Graphite (yield of modified Hummer's method, 35%)	0.324	0.0746	0.02

H_2SO_4	12.3	0.129	1.58
H_2O_2	1.29	0.168	0.22
HCL	34.9	0.0193	0.67
$KMnO_4$	1.62	0.104	0.17
Alumina	533	0.0637	33.96
Cu $(NO_3)_2$ $6H_2O$	36.2	0.135	4.89
H_2BDC	181	0.251	45.47
NMP	381	0.0611	23.28
PMMA	762	0.0676	5.15
NH_4OH	0.0161	0.100	0.00
DMF	3.62×10^{3}	0.0154	55.74
Acetonitrile	3.62×10^{3}	0.0122	44.00
Methanol	3.62×10^{3}	0.000268	0.97
Ethanol	3.62×10^{3}	0.0140	50.66
Total			226.78

The labour cost (C_{ml}, \$ m^{-2}) is calculated via Equation S6:

$$Cml = t \times C_{lh} \qquad \text{Equation S6}$$

where t (h) represents the working hours required for membrane fabrication, and C_{lh} (\$25.20 h^{-1}) denotes the hourly labour cost obtained from the Office for National Statistics (*103*). The total labour cost is calculated to be \$379.85. Accordingly, the membrane unit cost was \$646.63.

4.3.4 Pump cost, C_p

Two types of pumps are designed in the desalination process: circulation pumps and vacuum pumps.

The circulation pumps are employed to maintain continuous flow of the feed stream in the nanofiltration, pervaporation, and water carrier pervaporation processes, ensuring stable crossflow velocity across the membrane surfaces to enhance mass transfer and minimize

fouling. In addition, for the pervaporation and water carrier pervaporation systems, circulation pumps are also used to drive the cooling water loop on the permeate side, facilitating efficient condensation of the permeated vapour. Vacuum pumps are installed on the permeate side of the pervaporation process to generate and maintain a low-pressure environment.

Proper sizing of the pumps is therefore critical to ensure stable process performance and to optimize the overall energy efficiency of the separation system.

a) Circulating pumps

The pump power (E_{cp}, kW) was calculated as follows (*104*):

$$Ecp = \frac{vf \times \rho \times g \times h}{eta \times 1000} \quad \text{Equation S7}$$

where ρ (kg m^{-3}) indicates the liquid density, g (m s^{-2}) is the gravitational acceleration, h (m) refers to the pump head, and eta (%) is the pump efficiency assumed to be 80% in this study. vf represents the volumetric flow rate of the circulating stream (m³ s^{-1}) driven by the circulated pump. In this study, the plant was designed with a total capacity of 500 m³ day^{-1}, corresponding to an overall membrane flux of 5.787×10^{-3} m³ s^{-1}. Assuming the use of four circulation pumps to supply the plant, the velocity of each pump in the circulation loop was set to 1.45×10^{-2} m³ s^{-1} to ensure adequate crossflow and minimize fouling on the feeding side.

In the pervaporation and water carrier pervaporation processes, the required flow rate pervaporation in the cooling system was calculated based on the energy balance during the cooling process.

In the pervaporation process, the total heat ($Q_{totalPV}$, kW) to be removed is composed of both sensible heat and latent heat from a stream/vapour condensation(*105*).

$$Q_{total\_PV} = m_c \times C_p \times \Delta T_1 + m_c \times \lambda \quad \text{Equation S8}$$

where m_c (kg s^{-1}) refers to the mass flow rate of the fluid being cooled. C_p (kJ kg^{-1} K^{-1}) is the specific heat capacity of the cooling water. ΔT_1 (K) indicates the temperature change of the vapour before condensation. λ (kJ kg^{-1}) is the latent heat of condensation.

The heat absorbed by the cooling water is expressed by Equation S9.

$$Q_{absorbed\_PV} = \dot{m}_{cooling\_PV} \times C_p \times \Delta T_2 \quad \text{Equation S9}$$

where $\dot{m}_{cooling}$ (kg s^{-1}) is the mass flow rate of the coolant water and ΔT_2 (K) is the temperature rise of the coolant water.

By conserving energy, the mass flow rate of the cooling water can be calculated via:

$$\dot{m}_{cooling\_PV} = \frac{m_c \times C_p \times \Delta T_1 + m_c \times \lambda}{C_p \times \Delta T_2} \quad \text{Equation S10}$$

The volumetric flow rate of the coolant is subsequently calculated via Equation S11:

$$q_{cooling\_PV} = \frac{\dot{m}_{cooling}}{\rho}$$ Equation S11

In terms of water carrier pervaporation, the total heat (Q_{total_WCPV}, kW) can be calculated as follows:

$$Q_{total\_WCPV} = J \times A \times \lambda$$ Equation S12

where J (kg m^{-2} s^{-1}) is the permeate flux supported by each pump, and A (m^2) refers to the effective membrane area.

The volumetric flow rate of the coolant in the water carrier pervaporation process is estimated via Equation S13:

$$q_{cooling\_WCPV} = \frac{J \times A \times \lambda \times 1000}{C_p \times \rho \times \Delta T_3}$$ Equation S13

where ΔT_3 (K) is the temperature change on the permeate side.

The general cost correction of a circulate pump (C_{cp}, \$) is presented in Equation S14 (*106*).

$$C_{cp} = Log(Ecp) + a1 \times Ecp^2 + b1 \times Ecp + c1$$ Equation S14

The corresponding coefficients $a1$, $b1$, and $c1$ are adjusted based on the Turton correlations (*107*) and the cost correlations proposed by Feng and Rangaiah (*108*).

b) Vacuum pump

The cost of a vacuum pump is determined based on its vacuum capacity (V_{pc}, m^3 h^{-1}) (*109*).

$$q_{cooling\_WCPV} = \frac{\dot{m}c \times R \times Tp \times 3600}{MW \times Pin \times \mathcal{E}p}$$ Equation S15

where $\dot{m}c$ (kg s^{-1}) represents the mass flow rate of the non-condensable gas in the pervaporation process. For desalination, $\dot{m}c$ is typically assumed to be 0.5% of the water flow rate, falling within the generally accepted range of 0.1–1% for such systems (*110*). R (J mol^{-1} K^{-1}) refers to the universal gas constant. Tp (K) represents the temperature on the permeate side of the membrane. MW (g mol^{-1}) is the molecular weight of the non-condensable gas; $\mathcal{E}p$ refers to the pump efficiency. Pin (mbar) represents the pressure on the permeate side. The purchase cost of vacuum pumps of different capacities can be found in the capacity–cost correlations reported in the literature (*111*).

4.3.5 Heat exchanger, C_{he}

The thermal energy required to heat the feed in both pervaporation and water-carrier pervaporation processes is estimated via the heat balance equation (*112*):

$$Q_f = \Sigma \dot{m} \times C_{ps} \times \Delta T$$ Equation 16

where $\dot{m}$ (kg s^{-1}) denotes the mass flow rate of the saline water heated by the heat exchanger and C_{ps} (kj kg^{-1} K^{-1}) represents the specific heat capacity of the saline water ΔT (K)

corresponds to the temperature difference of the saline water stream before and after passing through the heat recovery exchanger.

In the cooling system, the energy Q_c (kW) required to compensate for the heat of the water vapour was determined by:

$$Q_c = \dot{m}_p \times \lambda \quad \text{Equation S17}$$

where $\dot{m}_p$ indicates the flow rate of the cooling water cooled by the heat exchanger.

The cost of heat exchangers is estimated based on the heat exchanger area (A_{he}, m^2), which is calculated via Equation S18.

$$A_{he} = \frac{Q}{U \times \Delta T_m} \quad \text{Equation S18}$$

where Q is the thermal duty, U (W m^{-2} K^{-1}) indicates the overall heat transfer coefficient, which is assumed to be 1,500 W m^{-2} K^{-1} for the condensation of pervaporation and 600 W m^{-2} K^{-1} for the heating of the feed solution as well as the cooling of the water carrier pervaporation process based on the Engineering Tool Box (*113*). ΔT_m is the logarithmic mean temperature difference calculated via Equation 19 (*114*).

$$\Delta T_m = \frac{(T_{hot,in} - T_{cold,out}) - (T_{hot,out} - T_{cold,in})}{\ln\left(\frac{(T_{hot,in} - T_{cold,out})}{(T_{hot,out} - T_{cold,in})}\right)} \quad \text{Equation S19}$$

where $T_{hot,in}$ and $T_{hot,out}$ are the inlet and outlet of the hot stream, respectively, while the $T_{cold,in}$ and $T_{cold,out}$ refer to the inlet and outlet of the cold stream, respectively. Based on the calculated heat exchange area, the cost of the heat exchanger is determined by following the cost model correlated based on the Matche's data and Feng and Rangaiah's (*115*) data for a shell-and-tube heat exchanger (*106*).

$$C_{he} = Log(A\_he) + a2 \times A\_he^2 + b2 \times A\_he + c2 \quad \text{Equation S20}$$

4.3.6 Installation of equipment, C_{ins}

The equipment installation cost C_{ins} is assumed to be 25% of the purchase cost of the system, in accordance with the typical range (20–60%) reported in Plant Design and Economics for Chemical Engineers(*116*).

$$C_{ins} = 0.25 \times (C_{pv} + C_m + C_p + C_{he}) \quad \text{Equation S21}$$

4.3.7 Total direct cost, D_c

The total direct cost comprises the costs of equipment, membrane fabrication, and installation.

$$D_c = C_{pv} + C_m + C_p + C_{he} + C_{ins} + C_{pt} \quad \text{Equation S22}$$

4.3.8 Indirect cost, ID_c

Indirect costs, including freight, insurance, and field supervision costs, are assumed to constitute 10% of the total direct cost(*114*).

4.3.9 Total capital cost, C_c

The total capital cost includes both direct and indirect components:

$$C_c = D_c + ID_c \qquad \text{Equation S23}$$

4.3.10 Annual capital cost, A_c

The annual capital cost is calculated using the capital recovery method(*117*):

$$A_c = a(D_c + ID_c) \qquad \text{Equation S24}$$

where a refers to the amortization factor calculated via Equation S25(*63*):

$$a = \frac{i(1+i)^n}{(1+i)^n - 1} \qquad \text{Equation S25}$$

where i and n refer to the interest rate and lifetime of the plant and are assumed to be 5% and 20 years, respectively.

4.4 OPEX

The annual operational cost encompasses all expenses incurred following plant commissioning and throughout its operation. This includes the costs of membrane replacement (A_{mr}, \$ year^{-1}), energy consumption of both steam (A_{st}, \$ year^{-1}) and electricity (A_{el}, \$ year^{-1}), equipment maintenance (A_{main}, \$ year^{-1}), labour (A_{lb}, \$ year^{-1}) and brine disposal (A_{br}, \$ year^{-1}).

4.4.1 Membrane replacement, A_{mr}

The membrane replacement rate (M_r, %) is the annual membrane replacement rate, ranging from 10% to 20% depending on salinity (*118*).

$$A_{mr} = M_r \times C_m \qquad \text{Equation S26}$$

4.4.2 Energy consumption

Energy consumption includes electricity (E_c, kW) for circulating pumps (E_{cp}, kW) and vacuum pumps (E_{vp}, kW), as well as steam for heating (T_c, kW).

a) Electricity

The electricity energy consumed by the feeding pump (E_{vp}, kW) is calculated via Equation S27 (*119*):

$$E_{vp} = \frac{m_c R T_p}{MW \varepsilon_{vp}} \frac{\varphi}{\varphi - 1} [\left(\frac{P_{out}}{P_{in}}\right)^{\frac{\varphi - 1}{\varphi}} - 1] \qquad \text{Equation S27}$$

where ε_{vp} is the efficiency of the vacuum pump (80%) and P_{out} refers to the exit pressure of the vacuum pump (1013.25 mbar). φ indicates the adiabatic expansion coefficient, which is defined as (*119*):

$$\varphi = \frac{C_{cp}}{C_{cp} - R_{air}} \qquad \text{Equation S28}$$

where C_{cp} and $C_{cp} - R_{air}$ denote the heat capacity of air at constant pressure (1 kJ kg^{-1}) and constant volume (0.718 kJ kg^{-1}), respectively.

The annual electricity cost can then be estimated via Equation S29:

$$A_{el} = E \times ce \times f \times 365 \times 24 \qquad \text{Equation S29}$$

where E (kWh) refers to the electricity energy consumed, ce (\$ kWh^{-1}) indicates the unit electricity price acquired from the U.S. Energy Information Administration (EIA) (*113*), and f is the plant capacity (0.9).

b) Steam

The feeding energy (kWh) provided by steam equals the energy used for the temperature used for heating the fresh provided feeding solution by T_1 (from room temperature to the desired feeding temperature) and the circulated liquid by T_2 (from the downstream temperature to the upstream temperature). Thus, it can be calculated via Equation S30:

$$T_S = \frac{vf \times C_{ps} \times T_2 \times Rr + vf \times T_1 \times (1 - Rr)}{\lambda s} \qquad \text{Equation S30}$$

where Rr refers to the membrane recovery ratio and λs (kJ kg-1) indicates the latent heat of saturated steam at physical pressure.

The steam cost (C_s, \$ year-1) is defined as follows:

$$A_{St} = T_s \times cs \times f \times 365 \times 24 \qquad \text{Equation S31}$$

Where cs (\$ kWh^{-1}) indicates the unit steam cost.

4.4.3 Maintenance

The cost for plant maintenance is proportional to the plant capacity expressed by (*120*):

$$A_{main} = E_{main} \times F \times f \times 365 \times \frac{CEPCI\_2024}{CEPCI\_2007} \qquad \text{Equation S32}$$

where the specific maintenance and spare costs are assumed to be 0.033 \$ m^{-3} (*63, 118*).

4.4.4 Labour

The labour cost is estimated based on the plant capacity and the specific cost of operating labour (E_{lb}, 0.05 \$ m$^{-3)}$ (*63*).

$$A_{lb} = E_{lb} \times F \times f \times 365 \times \frac{CEPCI_{2024}}{CEPCI_{2002}} \qquad \text{Equation S33}$$

4.4.5 Brine disposal

The specific cost of brine disposal is calculated via Equation S34:

$$A_{br} = E_{br} \times F \times f \times 365 \times \frac{CEPCI_{2024}}{CEPCI_{2000}} \quad \text{Equation S34}$$

where the brine disposal coefficient, E_{br} (0.0015 \$ m^{-3}) is estimated based on the Sethi & Wiesner cost (*118*).

4.4.6 Total annual operation cost

The total annual cost is calculated as the sum of all annual operational costs.

$$A_O = A_{mr} + A_{st} + A_{el} + A_{main} + A_{lb} + A_{br} \quad \text{Equation S35}$$

The complete sets of capital expenditures and operating costs calculated on the basis of the abovementioned theories are summarized in Table S8.

Table S8 Capital expenditures and operating costs of the RO, PV and WCPV processes

Cost	RO	PV	WCPV
Pipes and valves ($)	103,700	42,900	46,200
Pretreatment ($)	62,600	62,600	62,600
Membrane cost ($)	4,490,000	114,200	154,900
Circulated pump ($)	853,600	317,800	197,400
Vacuum pump ($)	/	2,064,000	/
Heat exchanger ($)	/	2,107,000	836,900
Installation cost ($)	1,362,000	1,161,000	308,800
Direct cost ($)	6,872,000	5,869,000	1,606,800
Indirect cost ($)	687,200	586,900	160,700
**CAPEX** ($)	**7,559,200**	**6,455,900**	**1,767,500**
**Annual capital cost ($/year)**	**606,600**	**518,100**	**141,800**
Membrane replacement ($/year)	898,100	22,800	31,000
Electricity ($/year)	78,400	153,400	22,400
Steam ($/year)	/	1,6000	1,300
Labour ($/year)	10,200	10,200	10,200
Brine disposal ($/year)	500	500	500
Maintenance ($/year)	8,500	8,500	8,500
**OPEX ($/year)**	**995,700**	**197,000**	**73,900**
**Total annual coast ($/year)**	**1,602,300**	**715,100**	**215,700**

Supplementary note 2: Rationale of selecting parameters for CuBDC synthesis.

The rationale for selection of important parameters: reaction time and temperature and concentrations of and are elucidated as below.

5.1 Reaction time.

The 24 h reaction time was deliberately selected to balance sufficient nucleation and growth with the maintenance of the advantageous laminar structure. Crystal formation is a kinetically and thermodynamically regulated process involving nucleation, coordination assembly, and lattice maturation (*121*). As shown in fig. S20A, and B, membranes prepared with shorter MOF growth times exhibit incomplete crystallization. Under these conditions, the resulting CuBDC domains display low crystallinity and poorly defined structures and may even contain amorphous regions (fig. S20B). Such immature frameworks typically possess higher defect densities and under-coordinated metal sites, which compromise structural stability and separation performance. In contrast, excessive reaction time promotes uncontrolled crystal growth, leading to overgrowth of CuBDC nanosheets on same part of membrane. After 36 h of growth (fig. S20C), the nanosheets begin to crowd adjacent crystals and extend in the out-of-plane direction. This structural evolution is consistent with the enhanced diffraction features observed in the XRD pattern (fig. S20A), indicating increased crystallite size and stacking. Notably, this out-of-plane growth is primarily confined to the membrane surface, while the lower regions largely retain their laminar structure, suggesting that confined growth within the interlayers is still maintained (fig. S20D). This is further supported by the presence of small, discrete nanosheets observed on the exfoliated membrane (fig. S20E). Nevertheless, surface overgrowth can deteriorate the structural integrity of the upper membrane layer and potentially disrupt transport pathways. Therefore, a growth duration of 24 h was optimized to achieve effective defect refinement and adequate crystallinity while preserving membrane integrity and avoiding excessive overgrowth.

5.2 Temperature.

The synthesis temperature was selected based on conclusions from a previous study (*35*), which demonstrated that increasing the reaction temperature led to the evolution of ultrathin nanosheets into thicker platelet-like structures. To preserve the ultrathin morphology and avoid excessive crystal thickening, the reaction was therefore conducted at room temperature rather than under elevated thermal conditions.

REFERENCES

1. M. Melucci, L. Bocchi, M. Zambianchi, V. Palermo, Graphene-based filters for customized drinking water purification. *Nat. Water* **3**, 369–371 (2025).

2. S. Pei, H.-M. Cheng, The reduction of graphene oxide. *Carbon* **50**, 3210–3228 (2012).

3. R. K. Joshi, P. Carbone, F. C. Wang, V. G. Kravets, Y. Su, I. V. Grigorieva, H. A. Wu, A. K. Geim, R. R. Nair, Precise and ultrafast molecular sieving through graphene oxide membranes. *Science* **343**, 752–754 (2014).

4. R. R. Nair, H. A. Wu, P. N. Jayaram, I. V. Grigorieva, A. K. Geim, Unimpeded permeation of water through helium-leak–tight graphene-based membranes. *Science* **335**, 442–444 (2012).

5. K. H. Thebo, X. Qian, Q. Zhang, L. Chen, H.-M. Cheng, W. Ren, Highly stable graphene-oxide-based membranes with superior permeability. *Nat. Commun.* **9**, 1486 (2018).

6. H. Liu, H. Wang, X. J. A. M. Zhang, Facile fabrication of freestanding ultrathin reduced graphene oxide membranes for water purification. *Adv. Mater.* **27**, 249–254 (2015).

7. W.-S. Hung, C.-H. Tsou, M. De Guzman, Q.-F. An, Y.-L. Liu, Y.-M. Zhang, C.-C. Hu, K.-R. Lee, J.-Y. Lai, Cross-linking with diamine monomers to prepare composite graphene oxide-framework membranes with varying d-spacing. *Chem. Mater.* **26**, 2983–2990 (2014).

8. L. Chen, G. Shi, J. Shen, B. Peng, B. Zhang, Y. Wang, F. Bian, J. Wang, D. Li, Z. Qian, G. Xu, G. Liu, J. Zeng, L. Zhang, Y. Yang, G. Zhou, M. Wu, W. Jin, J. Li, H. Fang, Ion sieving in graphene oxide membranes via cationic control of interlayer spacing. *Nature* **550**, 380–383 (2017).

9. K. Goh, W. Jiang, H. E. Karahan, S. Zhai, L. Wei, D. Yu, A. G. Fane, R. Wang, Y. J. A. F. M. Chen, All-carbon nanoarchitectures as high-performance separation membranes with superior stability. *Adv. Funct. Mater.* **25**, 7348–7359 (2015).

10. F. Moghadam, M. Zhai, T. Zouaoui, K. Li, Hybrid graphene oxide membranes with regulated water and ion permeation channels via functional materials. *Curr. Opin. Chem. Eng.* **40**, 100907 (2023).

11. D. Bradshaw, A. Garai, J. Huo, Metal–organic framework growth at functional interfaces: Thin films and composites for diverse applications. *Chem. Soc. Rev.* **41**, 2344–2381 (2012).

12. H. G. Alemayehu, C. Liu, J. Hou, J. Yang, M. Fang, Z. Tang, L. Li, Highly stable membrane comprising MOF nanosheets and graphene oxide for ultra-permeable nanofiltration. *J. Membr. Sci.* **62**, 120479 (2022).

13. Z. Wang, J. Zhu, S. Xu, Y. Zhang, B. Van der Bruggen, Graphene-like MOF nanosheets stabilize graphene oxide membranes enabling selective molecular sieving. *J. Membr. Sci.* **633**, 119397 (2021).

14. W.-H. Zhang, M.-J. Yin, Q. Zhao, C.-G. Jin, N. Wang, S. Ji, C. L. Ritt, M. Elimelech, Q.-F. An, Graphene oxide membranes with stable porous structure for ultrafast water transport. *Nat. Nanotechnol.* **16**, 337–343 (2021).

15. F. Moghadam, C. Zhang, Z. Li, J. Li, M. Zhai, K. Li, Structurally stable hollow-fiber-based porous graphene oxide membranes with improved rejection performance by selective patching of framework defects with metal–organic framework crystals. *ACS Appl. Mater. Interfaces* **17**, 1803–1812 (2025).

16. X. Zhang, H. Wang, R. Yao, L. Wang, P. Zhao, Y. Li, L. Wang, Q. Wang, Q. Lyu, Z. Fan, Growing ZIF-L seeds in graphene oxide interlayers towards synthesizing efficient cation sieving membranes. *J. Membr. Sci.* **701**, 122718 (2024).

17. J. Y. Chong, B. Wang, K. Li, Water transport through graphene oxide membranes: The roles of driving forces. *Chem. Commun.* **54**, 2554–2557 (2018).

18. C. G. Carson, K. Hardcastle, J. Schwartz, X. Liu, C. Hoffmann, R. A. Gerhardt, R. Tannenbaum, Synthesis and structure characterization of copper terephthalate metal–organic frameworks. *Eur. J. Inorg. Chem.* **2009**, 2338–2343 (2009).

19. T. Wu, F. Moghadam, K. Li, High-performance porous graphene oxide hollow fiber membranes with tailored pore sizes for water purification. *J. Membr. Sci.* **645**, 120216 (2022).

20. A. M. Varghese, K. S. K. Reddy, G. N. Karanikolos, An in-situ-grown Cu-BTC metal–organic framework / graphene oxide hybrid adsorbent for selective hydrogen storage at ambient temperature. *Ind. Eng. Chem. Res.* **61**, 6200–6213 (2022).

21. W. Li, Y. Zhang, P. Su, Z. Xu, G. Zhang, C. Shen, Q. Meng, Metal–organic framework channelled graphene composite membranes for H2/CO2 separation. *J. Mater. Chem. A* **4**, 18747–18752 (2016).

22. L.-H. Xu, Y. Li, S.-H. Li, M.-Y. Lv, Z.-P. Zhao, Space-confined growth of 2D MOF sheets between GO layers at room temperature for superior PDMS membrane-based ester/water separation. *J. Membr. Sci.* **656**, 120605 (2022).

23. S. Zheng, Q. Tu, M. Wang, J. J. Urban, B. Mi, Correlating interlayer spacing and separation capability of graphene oxide membranes in organic solvents. *ACS Nano* **14**, 6013–6023 (2020).

24. B. Venu, V. Shirisha, B. Vishali, G. Naresh, R. Kishore, I. Sreedhar, A. Venugopal, A Cu-BTC metal–organic framework (MOF) as an efficient heterogeneous catalyst for the aerobic oxidative synthesis of imines from primary amines under solvent free conditions. *New J. Chem.* **44**, 5972–5979 (2020).

25. V. Georgakilas, M. Otyepka, A. B. Bourlinos, V. Chandra, N. Kim, K. C. Kemp, P. Hobza, R. Zboril, K. S. Kim, Functionalization of graphene: Covalent and non-covalent approaches, derivatives and applications. *Chem. Rev.* **112**, 6156–6214 (2012).

26. A. A. Ensafi, S. E. Moosavifard, B. Rezaei, S. K. Kaverlavani, Engineering onion-like nanoporous CuCo2O4 hollow spheres derived from bimetal–organic frameworks for high-performance asymmetric supercapacitors. *J. Mater. Chem. A* **6**, 10497–10506 (2018).

27. G. Zhan, L. Fan, F. Zhao, Z. Huang, B. Chen, X. Yang, S.-f. Zhou, Fabrication of Ultrathin 2D Cu-BDC Nanosheets and the Derived Integrated MOF Nanocomposites. *Adv. Funct. Mater.* **29**, 1806720 (2019).

28. B. Mao, D. G. Calatayud, V. Mirabello, B. J. Hodges, J. A. R. Martins, S. W. Botchway, J. M. Mitchels, S. I. Pascu, Interactions between an aryl thioacetate-functionalized Zn(II) porphyrin and graphene oxide. *Adv. Funct. Mater.* **26**, 687–697 (2016).

29. F. Yang, M. Wu, Y. Wang, S. Ashtiani, H. Jiang, A GO-Induced assembly strategy to repair MOF nanosheet-based membrane for efficient H2/CO2 separation. *ACS Appl. Mater.* **11**, 990–997 (2019).

30. M. Sharma, S. Rani, D. K. Pathak, R. Bhatia, R. Kumar, I. Sameera, Temperature dependent Raman modes of reduced graphene oxide: Effect of anharmonicity, crystallite size and defects. *Carbon* **184**, 437–444 (2021).

31. I. Miccoli, F. Edler, H. Pfnür, C. Tegenkamp, The 100th anniversary of the four-point probe technique: The role of probe geometries in isotropic and anisotropic systems. *J. Phys. Condens. Matter* **27**, 223201 (2015).

32. N. F. D. Aba, J. Y. Chong, B. Wang, C. Mattevi, K. Li, Graphene oxide membranes on ceramic hollow fibers–Microstructural stability and nanofiltration performance. *J. Membr. Sci.* **484**, 87–94 (2015).

33. T. Yang, H. Lin, K. P. Loh, B. Jia, Fundamental transport mechanisms and advancements of graphene oxide membranes for molecular separation. *Chem. Mater.* **31**, 1829–1846 (2019).

34. N. Mahdjoub, N. Allen, P. Kelly, V. Vishnyakov, SEM and Raman study of thermally treated TiO2 anatase nanopowders: Influence of calcination on photocatalytic activity. *J. Photochem. Photobiol. A Chem.* **211**, 59–64 (2010).

35. T. Rodenas, I. Luz, G. Prieto, B. Seoane, H. Miro, A. Corma, F. Kapteijn, F. X. Llabrés i Xamena, J. Gascon, Metal–organic framework nanosheets in polymer composite materials for gas separation. *Nat. Mater.* **14**, 48–55 (2015).

36. M. Zhai, H. Peng, K. Li, High-performance loose nanofiltration membranes with excellent antifouling properties for dye/salt separation. *J. Membr. Sci.* **708**, 123028 (2024).

37. H. Peng, K. Li, Nanostructured membranes with interconnected pores via a combination of phase inversion and solvent crystallisation approach. *J. Membr. Sci.* **680**, 121738 (2023).

38. F. Yang, J. Guo, C. Han, J. Huang, Z. Zhou, S.-P. Sun, Y. Zhang, L. Shao, Turing covalent organic framework membranes via heterogeneous nucleation synthesis for organic solvent nanofiltration. *Sci. Adv.* **10**, eadr9260 (2024).

39. P. Song, G. Natale, J. Wang, T. Bond, H. Hejazi, H. de la Hoz Siegler, I. Gates, Q. Lu, 2D and 3D metal–organic framework at the oil/water interface: A case study of copper benzenedicarboxylate. *Adv. Funct. Mater.* **6**, 1801139 (2019).

40. J. Wang, P. Zhang, B. Liang, Y. Liu, T. Xu, L. Wang, B. Cao, K. Pan, Graphene oxide as an effective barrier on a porous nanofibrous membrane for water treatment. *ACS Appl. Mater. Interfaces* **8**, 6211–6218 (2016).

41. I. N. Floros, E. P. Kouvelos, G. I. Pilatos, E. P. Hadjigeorgiou, A. D. Gotzias, E. P. Favvas, A. A. Sapalidis, Enhancement of flux performance in PTFE membranes for direct contact membrane distillation. *Membranes* **12**, 345 (2020).

42. S. Y. Park, J. W. Chung, S.-Y. Kwak, Regenerable anti-fouling active PTFE membrane with thermo-reversible "peel-and-stick" hydrophilic layer. *J. Membr. Sci.* **491**, 1–9 (2015).

43. S. Azizighannad, S. Mitra, Stepwise reduction of graphene oxide (GO) and its effects on chemical and colloidal properties. *Sci. Rep.* **8**, 10083 (2018).

44. Z. S. Tai, M. H. Abd Aziz, M. H. D. Othman, M. I. H. Mohamed Dzahir, N. A. Hashim, K. N. Koo, S. K. Hubadillah, A. F. Ismail, M. A. Rahman, J. Jaafar, Ceramic membrane distillation for desalination. *Sep. Purif. Rev.* **49**, 317–356 (2020).

45. X. Mu, X. Wu, T. Zhang, D. B. Go, T. Luo, Thermal transport in graphene oxide–From ballistic extreme to amorphous limit. *Sci. Rep.* **4**, 3909 (2014).

46. S. Alharbi, J. C. Crepeau, B. Rezaie, K. Kumar, Temperature dependence of thermal conductivity for water using the transient hot-wire method. *J. Engin. Thermophys.* **31**, 78–97 (2022).

47. H. Liu, Z. Huang, K. Liu, X. Hu, J. Zhou, Interfacial solar-to-heat conversion for desalination. *Adv. Energy Mater.* **9**, 1900310 (2019).

48. S. Zhao, C. Jiang, J. Fan, S. Hong, P. Mei, R. Yao, Y. Liu, S. Zhang, H. Li, H. Zhang, C. Sun, Z. Guo, P. Shao, Y. Zhu, J. Zhang, L. Guo, Y. Ma, J. Zhang, X. Feng, F. Wang, H. Wu, B. Wang, Hydrophilicity gradient in covalent organic frameworks for membrane distillation. *Nat. Mater.* **20**, 1551–1558 (2021).

49. Q. An, F. Li, Y. Ji, H. Chen, Influence of polyvinyl alcohol on the surface morphology, separation and anti-fouling performance of the composite polyamide nanofiltration membranes. *J. Membr. Sci.* **367**, 158–165 (2011).

50. Y. Okamoto, J. H. Lienhard, How RO membrane permeability and other performance factors affect process cost and energy use: A review. *Desalination* **470**, 114064 (2019).

51. Y. Mao, Q. Huang, B. Meng, K. Zhou, G. Liu, A. Gugliuzza, E. Drioli, W. Jin, Roughness-enhanced hydrophobic graphene oxide membrane for water desalination via membrane distillation. *J. Membr. Sci.* **611**, 118364 (2020).

52. S. Al-Gharabli, Z. Abu El-Rub, E. Hamad, W. Kujawski, Z. Flanc, K. Pianka, J. Kujawa, Surfaces with adjustable features-effective and durable materials for water desalination. *Int. J. Mol. Sci.* **22**, 11743 (2021).

53. K.-K. Yan, L. Jiao, S. Lin, X. Ji, Y. Lu, L. J. D. Zhang, Superhydrophobic electrospun nanofiber membrane coated by carbon nanotubes network for membrane distillation. *Desalination* **437**, 26–33 (2018).

54. M. S. El-Bourawi, Z. Ding, R. Ma, M. Khayet, A framework for better understanding membrane distillation separation process. *J. Membr. Sci.* **285**, 4–29 (2006).

55. K. W. Lawson, D. R. Lloyd, Membrane distillation. *J. Membr. Sci.* **124**, 1–25 (1997).

56. J. D. Seader, E. J. Henley, D. K. Roper, *Separation process principles: With applications using process simulators* (John Wiley & Sons, 2016).

57. C. Charcosset, *Membrane Processes in Biotechnology and Pharmaceutics* (Elsevier, 2012), 350 pp.

58. Y. Li, E. R. Thomas, M. H. Molina, S. Mann, W. S. Walker, M. L. Lind, F. Perreault, Desalination by membrane pervaporation: A review. *Desalination* **547**, 116223 (2023).

59. W. Kujawski, Application of pervaporation and vapor permeation in environmental protection. *Pol. J. Environ. Stud.* **9**, 13–26 (2000).

60. H. S. Usman, K. Touati, M. S. Rahaman, An economic evaluation of renewable energy-powered membrane distillation for desalination of brackish water. *Renew. Energy* **169**, 1294–1304 (2021).

61. U.S. Environmental Protection Agency (EPA), *2018 Edition of the Drinking Water Standards and Health Advisories* (Office of Water, EPA 822-F-18-001, 2018).

62. L. N. Nthunya, M. F. Bopape, O. T. Mahlangu, B. B. Mamba, B. Van der Bruggen, C. A. Quist-Jensen, H. Richards, Fouling, performance and cost analysis of membrane-based water desalination technologies: A critical review. *J. Environ. Manage.* **301**, 113922 (2022).

63. M.-C. Sparenberg, I. R. Salmón, P. Luis, Economic evaluation of salt recovery from wastewater via membrane distillation-crystallization. *J. Environ. Manage.* **235**, 116075 (2020).

64. M. Lee, "Micro-channel enhanced alumina membranes: Designing and tailoring their properties for widened applications", thesis, Imperial College London (2016).

65. J. Sun, X. Qian, Z. Wang, F. Zeng, H. Bai, N. Li, Tailoring the microstructure of poly(vinyl alcohol)-intercalated graphene oxide membranes for enhanced desalination performance of high-salinity water by pervaporation. *J. Membr. Sci.* **599**, 117838 (2020).

66. A. Huang, B. Feng, Synthesis of novel graphene oxide-polyimide hollow fiber membranes for seawater desalination. *J. Membr. Sci.* **548**, 59–65 (2018).

67. B. Liang, W. Zhan, G. Qi, S. Lin, Q. Nan, Y. Liu, B. Cao, K. Pan, High performance graphene oxide/polyacrylonitrile composite pervaporation membranes for desalination applications. *J. Mater. Chem. A* **3**, 5140–5147 (2015).

68. Y. Song, R. Li, F. Pan, Z. He, H. Yang, Y. Li, L. Yang, M. Wang, H. Wang, Z. Jiang, Ultrapermeable graphene oxide membranes with tunable interlayer distances via vein-like supramolecular dendrimers. *J.Mater. Chem. A* **7**, 18642–18652 (2019).

69. Y. Qian, X. Zhang, C. Liu, C. Zhou, A. Huang, Tuning interlayer spacing of graphene oxide membranes with enhanced desalination performance. *Desalination* **460**, 56–63 (2019).

70. B. Feng, K. Xu, A. Huang, Synthesis of graphene oxide/polyimide mixed matrix membranes for desalination. *RSC Adv.* **7**, 2211–2217 (2017).

71. X. Zhao, Z. Tong, X. Liu, J. Wang, B. Zhang, Facile preparation of polyamide–graphene oxide composite membranes for upgrading pervaporation desalination performances of hypersaline solutions. *Ind. Eng. Chem. Res.* **59**, 12232–12238 (2020).

72. X. Qian, N. Li, Q. Wang, S. Ji, Chitosan/graphene oxide mixed matrix membrane with enhanced water permeability for high-salinity water desalination by pervaporation. *Desalination* **438**, 83–96 (2018).

73. F. Ugur Nigiz, Graphene oxide-sodium alginate membrane for seawater desalination through pervaporation. *Desalination* **485**, 114465 (2020).

74. E. Halakoo, X. Feng, Layer-by-layer assembly of polyethyleneimine/graphene oxide membranes for desalination of high-salinity water via pervaporation. *Sep. Purif. Technol.* **234**, 116077 (2020).

75. Z. Wang, J. Sun, N. Li, Y. Qin, X. Qian, Z. Xie, Tuning interlayer structure to construct steady dual-crosslinked graphene oxide membranes for desalination of hypersaline brine via pervaporation. *Sep. Purif. Technol.* **286**, 120459 (2022).

76. D. D. Kachhadiya, Z. V. P. Murthy, Graphene oxide modified CuBTC incorporated PVDF membranes for saltwater desalination via pervaporation. *Sep. Purif. Technol.* **290**, 120888 (2022).

77. Y. Liu, Z. Tong, H. Zhu, X. Zhao, J. Du, B. Zhang, Polyamide composite membranes sandwiched with modified carbon nanotubes for high throughput pervaporation desalination of hypersaline solutions. *J. Membr. Sci.* **641**, 119889 (2022).

78. G. Liu, J. Shen, Q. Liu, G. Liu, J. Xiong, J. Yang, W. Jin, Ultrathin two-dimensional MXene membrane for pervaporation desalination. *J. Membr. Sci.* **548**, 548–558 (2018).

79. Y. Zhang, H. Yuan, X. Chen, Z. Jiang, J. Lu, F. Xin, Incorporating MXene@MOF-303 composites into Poly(vinyl alcohol) (PVA) to fabricate pervaporation membranes for desalination. *ACS Appl. Polym. Mater.* **6**, 8277–8290 (2024).

80. D. Unlu, Water desalination by pervaporation using MIL-101(Cr) and MIL-101(Cr)@ GODoped PVA hybrid membranes. *Water Air Soil Pollut.* **234**, 96 (2023).

81. Z. Zeng, H. Yuan, J. Lu, Preparation and characterization of PVA-g-GO/PVA/PAN composite membrane for pervaporation desalination. *Can. J. Chem. Eng.* **101**, 2199–2211 (2023).

82. D. Qin, R. Zhang, B. Cao, P. Li, Fabrication of high-performance composite membranes based on hierarchically structured electrospun nanofiber substrates for pervaporation desalination. *J. Membr. Sci.* **638**, 119672 (2021).

83. Z. Li, J. Fan, L. Wang, X. Yang, L. Guo, H. Chen, D. Gong, G. Yang, Q. Xu, S. Zou, G. Zeng, Two-dimensional lamellar stacking COF membrane with charge repulsion effect for ions separation. *J. Membr. Sci.* **699**, 122645 (2024).

84. G. Yang, D. Ng, Z. Huang, J. Zhang, S. Gray, Z. Xie, Janus hollow fibre membranes with intrusion anchored structure for robust desalination and leachate treatment in direct contact membrane distillation. *Desalination* **551**, 116423 (2023).

85. Z. Zhu, M. Song, F. Qu, Y. Zhou, Y. Yang, J. Qi, J. Li, Engineering multinanochannel polymer-intercalated graphene oxide membrane for strict volatile sieving in membrane distillation. *Environ. Sci. Technol.* **58**, 1399–1409 (2024).

86. A. B. Yeszhanov, I. V. Korolkov, O. Güven, G. B. Melnikova, S. S. Dosmagambetova, A. N. Borissenko, A. K. Nurkassimov, M. T. Kassymzhanov, M. V. Zdorovets, Effect of hydrophobized PET TeMs membrane pore-size on saline water treatment by direct contact membrane distillation. *RSC Adv.* **14**, 4034–4042 (2024).

87. W. Zhang, Y. Lu, J. Liu, X. Li, B. Li, S. Wang, Preparation of re-entrant and anti-fouling PVDF composite membrane with omniphobicity for membrane distillation. *J. Membr. Sci.* **595**, 117563 (2020).

88. H. Feng, H. Li, M. Li, X. Zhang, Construction of omniphobic PVDF membranes for membrane distillation: Investigating the role of dimension, morphology, and coating technology of silica nanoparticles. *Desalination* **525**, 115498 (2022).

89. N. G. P. Chew, S. Zhao, C. Malde, R. Wang, Superoleophobic surface modification for robust membrane distillation performance. *J. Membr. Sci.* **541**, 162–173 (2017).

90. J. E. Efome, D. Rana, T. Matsuura, F. Yang, Y. Cong, C. Q. Lan, Triple-layered nanofibrous metal–organic framework-based membranes for desalination by direct contact membrane distillation. *ACS Sustain. Chem. Eng.* **8**, 6601–6610 (2020).

91. L. Chen, F. Li, L. Jiang, F. He, Y. Wei, UiO-66-NH2/PVA composite Janus membrane with a dense hydrophilic surface layer for strong resistance to fouling and wettability in membrane distillation. *J. Water Process Eng.* **48**, 102887 (2022).

92. D. Hou, Z. Wang, K. Wang, J. Wang, S. Lin, Composite membrane with electrospun multiscale-textured surface for robust oil-fouling resistance in membrane distillation. *J. Membr. Sci.* **546**, 179–187 (2018).

93. M. Essalhi, M. Khayet, S. Tesfalidet, M. Alsultan, N. Tavajohi, Desalination by direct contact membrane distillation using mixed matrix electrospun nanofibrous membranes with carbon-based nanofillers: A strategic improvement. *Chem. Eng. J.* **426**, 131316 (2021).

94. S. Munirasu, F. Banat, A. A. Durrani, M. A. Haija, Intrinsically superhydrophobic PVDF membrane by phase inversion for membrane distillation. *Desalination* **417**, 77–86 (2017).

95. T. Pan, J. Liu, N. Deng, Z. Li, L. Wang, Z. Xia, J. Fan, Y. Liu, ZnO Nanowires@PVDF nanofiber membrane with superhydrophobicity for enhanced anti-wetting and anti-scaling properties in membrane distillation. *J. Membr. Sci.* **621**, 118877 (2021).

96. M. M. Sayed, H. M. Mousa, A. H. El-Shazly, A. Zkria, T. Yoshitake, M. ElKady, Novel post-heat treatment green biodegradable PLA@SiO2 nanocomposite membrane for water desalination. *J. Environ. Chem. Eng.* **12**, 114378 (2024).

97. Y. Zhang, J. Y. Chong, Y. Zhao, R. Xu, A. Asakawa, R. Wang, Facile hydrophobic modification of hydrophilic membranes by fluoropolymer coating for direct contact membrane distillation. *J. Membr. Sci.* **672**, 121432 (2023).

98. J. Ju, K. Fejjari, Y. Cheng, M. Liu, Z. Li, W. Kang, Y. Liao, Engineering hierarchically structured superhydrophobic PTFE/POSS nanofibrous membranes for membrane distillation. *Desalination* **486**, 114481 (2020).

99. Z. Huang, G. Yang, J. Zhang, S. Gray, Z. Xie, Dual-layer membranes with a thin film hydrophilic MOF/PVA nanocomposite for enhanced antiwetting property in membrane distillation. *Desalination* **518**, 115268 (2021).

100. L. F. Greenlee, D. F. Lawler, B. D. Freeman, B. Marrot, P. Moulin, Reverse osmosis desalination: Water sources, technology, and today's challenges. *Water Res.* **43**, 2317–2348 (2009).

101. K. K. Sirkar, L. Song, *Pilot-scale studies for direct contact membrane distillation-based desalination process* (US Department of the Interior, Bureau of Reclamation, 2009).

102. S. Sethi, M. R. Wiesner, Simulated cost comparisons of hollow-fiber and integrated nanofiltration configurations. *Water Res.* **34**, 2589–2597 (2000).

103. S. Jenkins, Economic Indicators: November 2025 (Editor's note about disruptions from U.S. government shutdown). Chemical Engineering (27 October 2025); www.chemengonline.com/economic-indicators-november-2025-editors-note-about-disruptions-from-u-s-government-shutdown/.

104. J. Yu, T. Zhang, J. Qian, Efficiency testing methods for centrifugal pumps. *Electr. Mot. Prod*, 125–172 (2011).

105. C. Geankoplis, *Transport processes and separation process principles* (*includes unit operations*) (Prentice Hall Press, 2003).

106. M. Shamoushaki, P. H. Niknam, L. Talluri, G. Manfrida, D. Fiaschi, Development of cost correlations for the economic assessment of power plant equipment. *Energies* **14**, 2665 (2021).

107. R. Turton, R. C. Bailie, W. B. Whiting, J. A. Shaeiwitz, *Analysis, Synthesis, and Design of Chemical Processes* (Pearson Education, ed. 3, 2008).

108. Y. Feng, G. P. Rangaiah, Evaluating capital cost estimation programs. *Chem. Eng.* **118**, (2011).

109. H. Perry Robert, W. Green Don, O. Maloney James, *Perry's chemical engineers' handbook* (Mc Graw-Hills, ed. 7, 1997), pp. 56-64.

110. R. Y. M. Huang, *Pervaporation Membrane Separation Processes* (Elsevier, 1991).

111. H. Loh, J. Lyons, C. W. White, "Process equipment cost estimation, final report" (National Energy Technology Lab, 2001).

112. A. Martin, O. Dahl, Techno-economic system analysis of membrane distillation process for treatment of chemical mechanical planarization wastewater in nano-electronics industries. *Sep. Purif. Technol.* **248**, 117013 (2020).

113. U.S. Energy Information Administration, “Electric Power Monthly, Table 5.6.A. Average Price of Electricity to Ultimate Customers by End-Use Sector, by State, December 2025 and 2024” (2026); www.eia.gov/electricity/monthly/epm_table_grapher.php?t=epmt_5_6_a.

114. I. Prihatiningtyas, A.-H. A. H. Al-Kebsi, Y. Hartanto, T. M. Zewdie, B. Van der Bruggen, Techno-economic assessment of pervaporation desalination of hypersaline water. *Desalination* **527**, 115538 (2022).

115. G. Towler, R. Sinnott, *Chemical engineering design: Principles, practice and economics of plant and process design* (Butterworth-Heinemann, 2021).

116. J. I. Peters, Review of: “ Plant Design And Economics For Chemical Engineers” Max S. Peters: McGraw-Hill Book Company, New York, 1958. 511 pp. $11.00. *Eng. Econ* **5**, 27–30 (1959).

117. F. Banat, N. Jwaied, Economic evaluation of desalination by small-scale autonomous solar-powered membrane distillation units. *Desalination* **220**, 566–573 (2008).

118. H. M. Ettouney, H. T. El-Dessouky, R. S. Faibish, P. J. Gowin, Evaluating the economics of desalination. *Chem. Eng. Prog.* **98**, 32–39 (2002).

119. Z. Xie, D. Ng, M. Hoang, S. Adnan, J. Zhang, M. Duke, J.-D. Li, A. Groth, C. Tun, S. Gray, Preliminary evaluation for vacuum membrane distillation (VMD) energy requirement. *J. Membr. Sci. Res.* **2**, 207–213 (2016).

120. A. Helal, A. El-Nashar, E. Al-Katheeri, S. Al-Malek, Optimal design of hybrid RO/MSF desalination plants Part I: Modeling and algorithms. *Desalination* **154**, 43–66 (2003).

121. H. Furukawa, K. E. Cordova, M. O’Keeffe, O. M. Yaghi, The Chemistry and applications of metal-organic frameworks. *Science* **341**, 1230444 (2013).
